# Supplementary material for: Drug GRADE: An Integrated Analysis of Population Growth and Cell Death Reveals Drug-Specific and Cancer Subtype-Specific Response Profiles
Source: Cell Rep. Author manuscript; Available in PMC 2020 Jul 31. (PMC7394473; doi:10.1016/j.celrep.2020.107800)
Supplement: 1 [file NIHMS1607445-supplement-1.pdf]

Cell Reports, Volume *31*

Supplemental Information

Drug GRADE: An Integrated Analysis of Population Growth and Cell Death Reveals Drug-Specific and Cancer Subtype-Specific Response Profiles

Hannah R. Schwartz, Ryan Richards, Rachel E. Fontana, Anna J. Joyce, Megan E. Honeywell, and Michael J. Lee

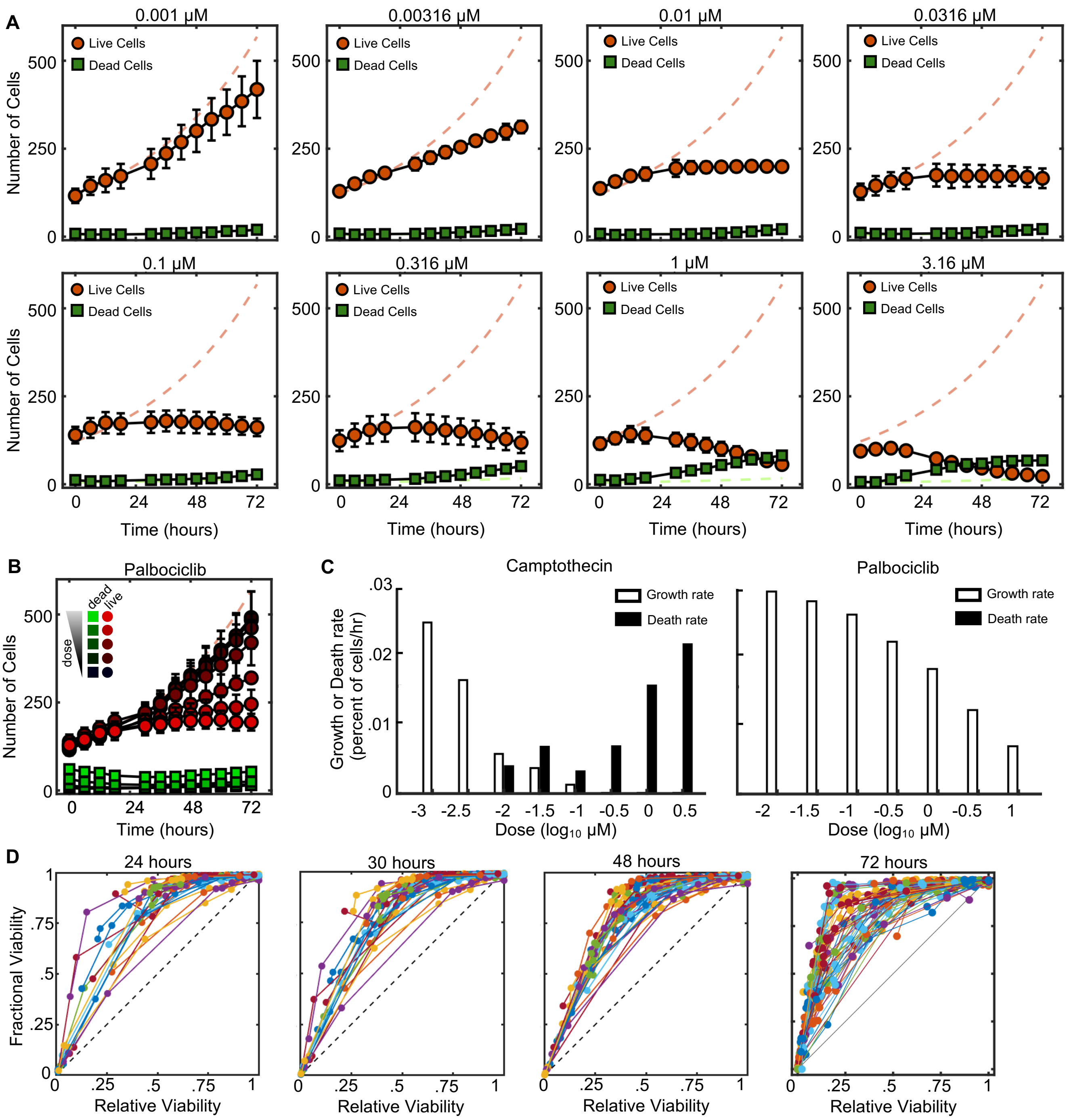

Figure S1: The relationship between relative and fractional responses differ by drug, Related to Figure 1. (A) The number of live and dead cells present over a 72-hour time course at different concentrations of Camptothecin, a bi-phasic drug. Dashed red and green are live and dead cell numbers for control untreated cells, respectively. **(B)** The number of live and dead cells present over a 72-hour time course at different concentrations of Palbociclib (10, 3.16, 1, 0.316, 0.1, 0.0316, or 0.01 μM). For (A) and (B) data are mean +/- s.d. of four replicates. **(C)** The growth rate and death rate calculated using an exponential growth model or lag-exponential death model, respectively, for each dose of Camptothecin and Palbociclib shown in panels (A) and (B). **(D)** RV and FV measured for a panel of 85 drugs at different time points as indicated. See also Table S1.

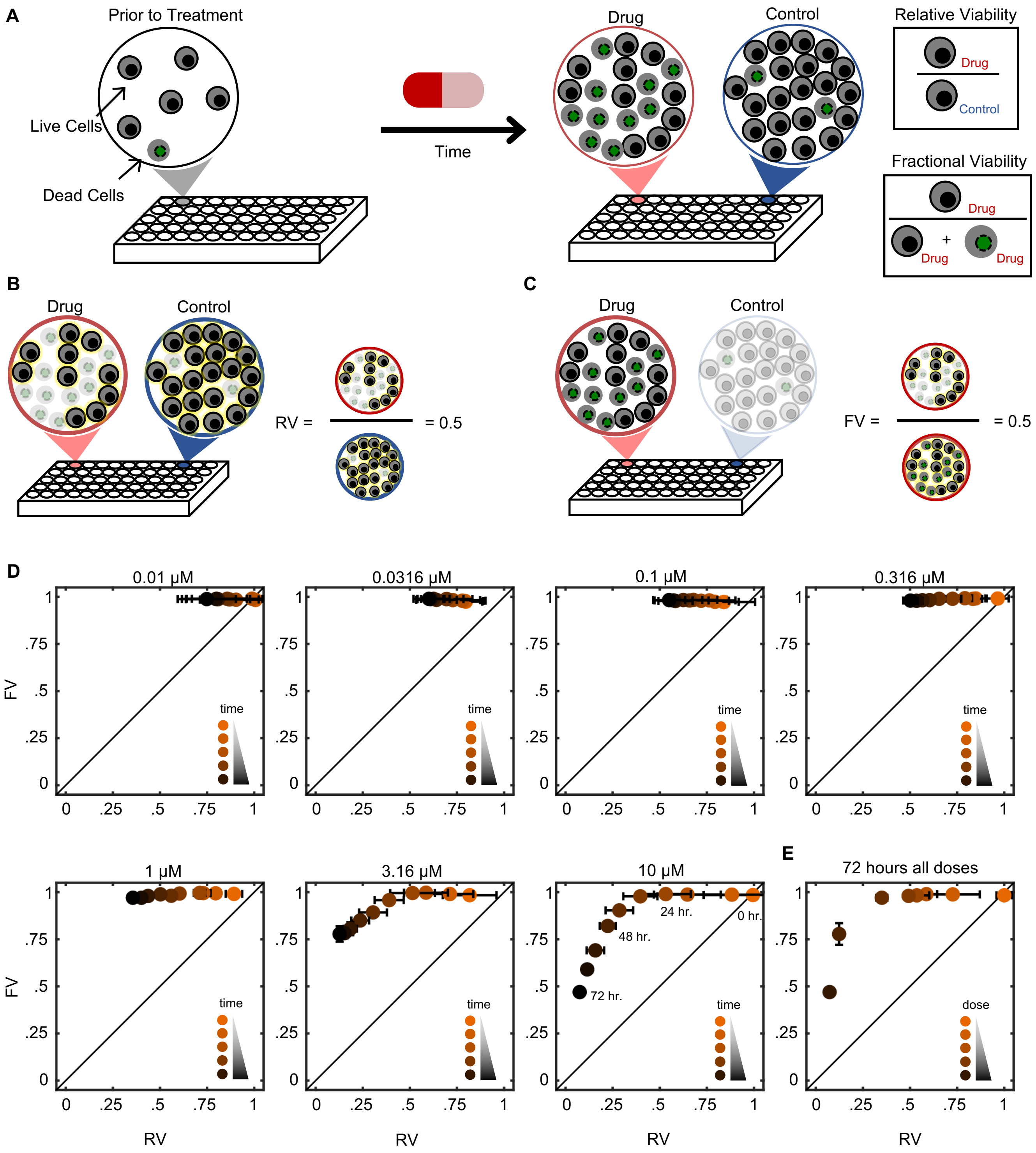

Figure S2: Relationship between RV50 and FV50, Related to Figure 2. (A) Schematic of a typical drug response assay in multi-well plates. Relative Viability (RV) and Fractional Viability (FV) are two common measures of response. **(B)** RV calculated for the example shown in (A). The example shown represents the IC50 of the RV measure (i.e. RV50), defined as the dose at which the observed number of live cells after drug exposure is half the size of the untreated population. **(C)** FV calculated for the example shown in (A). The example shown represents the IC50 of the FV measure (i.e. FV50), defined as the dose at which the population is half alive and half dead. Note, although both values in (B) and (C) are 0.5, these are computed from different comparisons and are defined differently. **(D)** RV/FV plots for each dose of Abemaciclib tested over a 72-hour time course using the STACK assay. Images were acquired every 8 hours. **(E)** RV versus FV plot for varied doses 72-hours after drug exposure. For panels (D) and E) data are mean +/- s.d. of two replicates.

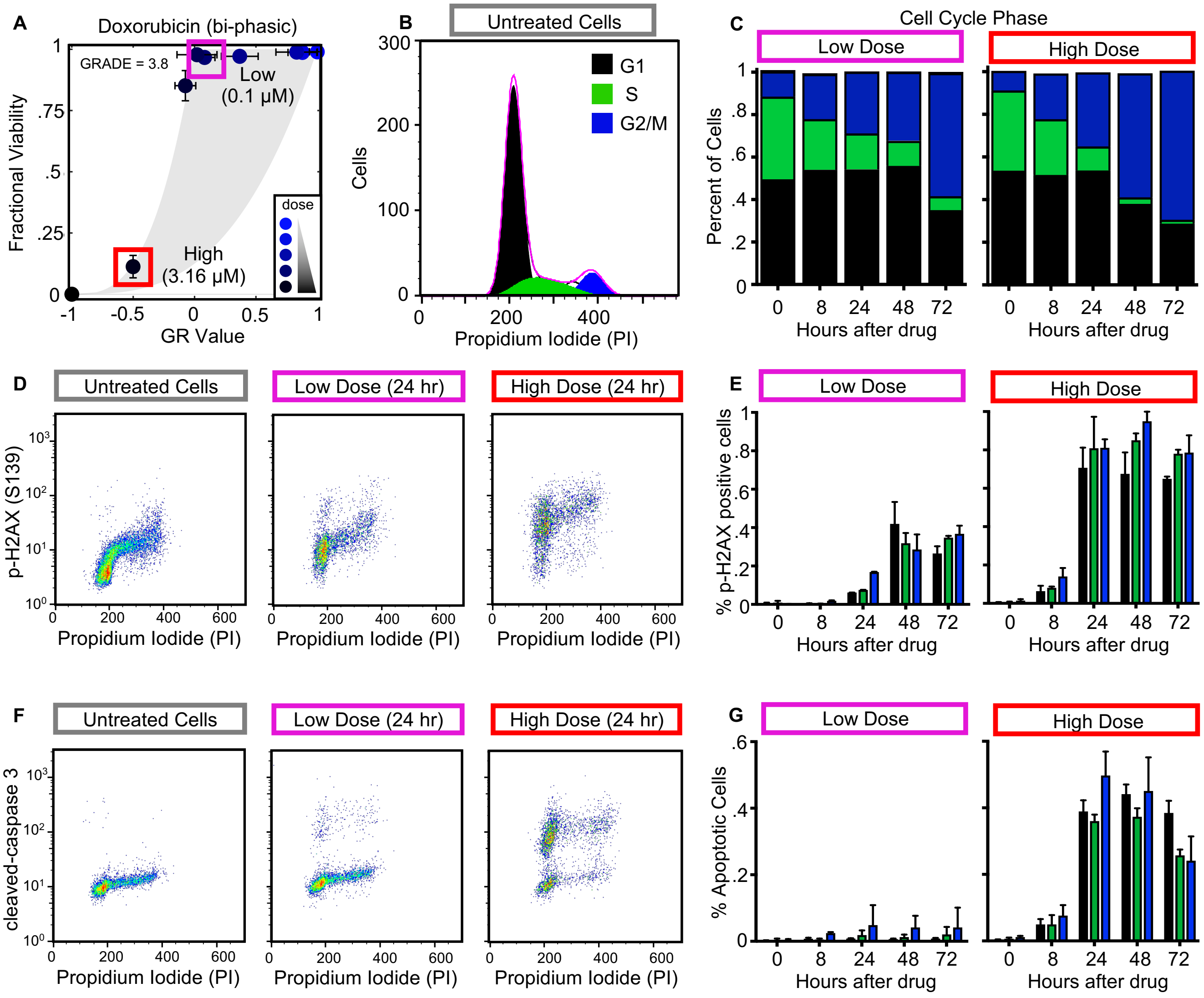

Figure S3: DNA damaging agents induce a bi-phasic drug response, Related to Figure 3. (A) Drug GR/FV plot for doxorubicin (DOX). Two doses highlighted: 0.1 µM (Low), which induces growth arrest, and 3.16 µM (High), which induces cell death but only following growth arrest. Data are mean +/- s.d. of 3 replicates. **(B-C)** Cell cycle analysis by flow cytometry using propidium iodide staining (PI). (B) Representative data for untreated cells. Cell cycle phase as determined by Dean-Jett-Fox algorithm shown. Data are representative of 3 biological replicates. (C) Cell cycle analysis for cells treated with Low or High dose DOX for indicated times. Colors are as in panel (B). **(D-E)** Analysis of DNA damage levels by flow cytometry. DNA double stranded breaks tracked using phosphorylation of H2AX (p-H2AX) in combination with cell cycle analysis using PI. (D) Representative data for untreated cells or cells exposed to Low or High dose DOX for 24 hours. Data are representative of 3 biological replicates. (E) H2AX phosphorylation kinetics for Low and High dose DOX. % H2AX positive cells determine by flow cytometry as in panel (D). Data are separated by cell cycle phase as in panels (B-C). Data are mean +/- s.d. of 3 biological replicates. **(F-G)** Flow cytometry-based analysis of apoptotic death in combination with cell cycle analysis using PI. (F) Representative data for untreated cells or cells exposed to Low or High dose DOX for 24 hours. Data are representative of 3 biological replicates. (G) Percentage of cleaved-caspase-3 positive cells measured using flow cytometry. Data are separated by cell cycle phase as in panels (B-C). Data are mean +/- s.d. of 3 biological replicates.

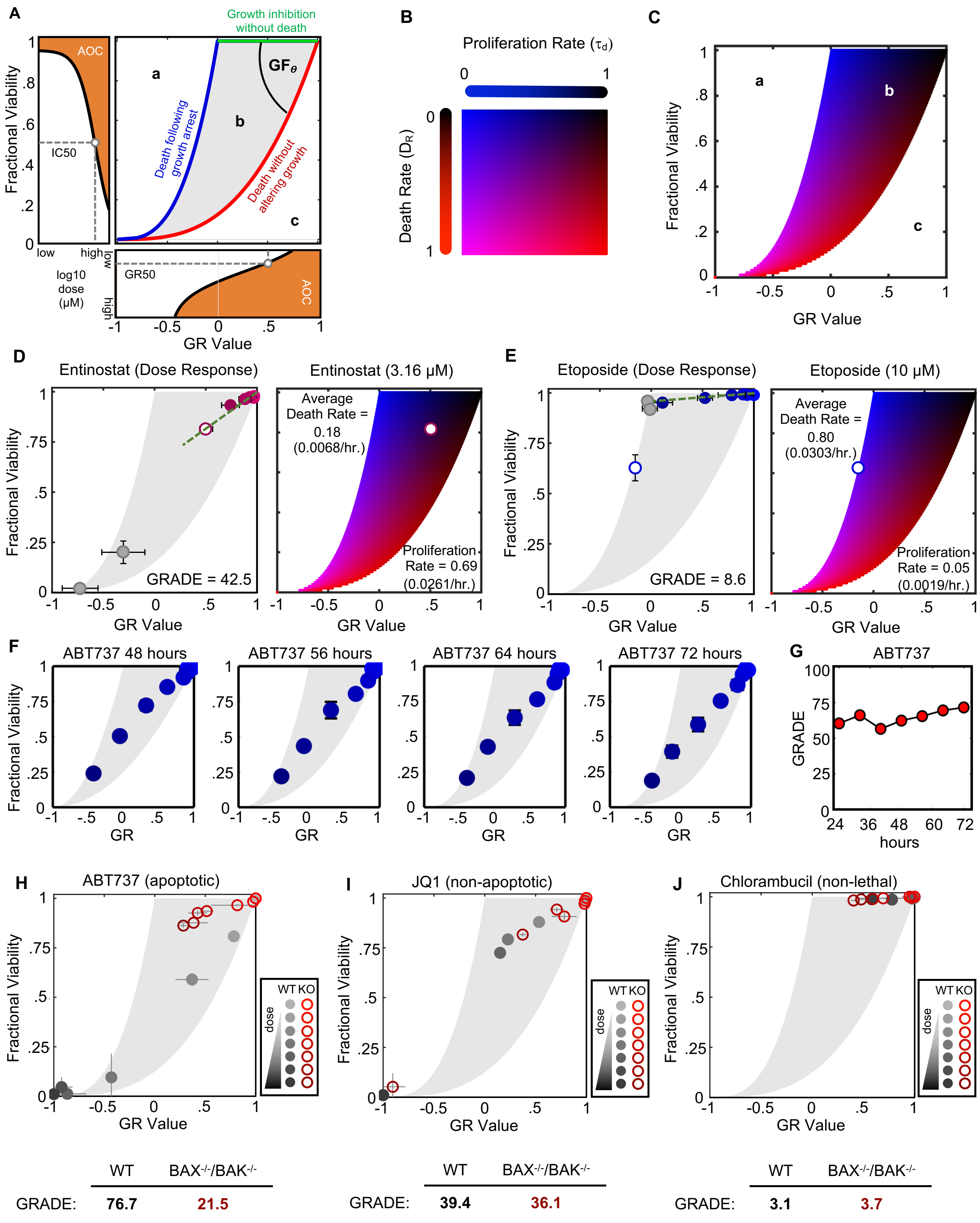

Figure S4: Stability and robustness of drug GRADE, Related to Figure 4. (A) Schematic of the GR/FV plot with reference limits shown for growth inhibition without death (green), death only following growth arrest (blue), and death without altering growth rate (red). Shaded region 'b' represents intermediate states in which a drug induces some growth inhibition and some death. **(B-C)** Simulated drug responses using all possible variations of drug-induced growth rate and death rates. **Figure legend continued on next page.**

Figure S4: Stability and robustness of drug GRADE, Related to Figure 4 (continued from previous page). (B) Color map of parameter values. Red increases as death rate increases. Blue increases as growth rate decreases. The scale for death rate and growth rate are relative to untreated growth rate. (**C)** FV and GR calculated for full parameter space in (B). **(D-E)** Examples of GRADE calculations (left) and inference of growth and death rates at individual doses (right). For each drug, doses not used in GRADE calculation are colored grey. White dot in each is a dose for which growth and death rates are highlighted. Growth rates and death rates are computed relative to the untreated cell proliferation rate. In these data the doubling time was 26.3868 hours (0.0379 cell divisions per hour). Data in (D-E) are mean +/- s.d. of 3 biological replicates. **(F-G)** GRADE stability of time. **(F)** FV vs. GR over time for ABT-737 (ABT). Data are mean +/- s.d. of four biological replicates. **(G)** Drug GRADE for ABT measured at time points listed. **(H-J)** FV and GR drug responses calculated for U2OS cells (WT, grey) or U2OS-$BAX^{-/-}/BAK^{-/-}$ (KO, red). BAX/BAK double knockout renders cells resistant to drugs that induce apoptotic death. Data are from Richards et al. (2020). Dose of drug represented by increasing shades of grey or red. Data are are the mean +/- s.d. of biological triplicate measurements **(H)** BH3 mimetic, ABT737, induces apoptotic death in U2OS cells. **(B)** BRD4/Bromodomain inhibitor, JQ1, induces non-apoptotic death in U2OS cells. **(C)** Nitrogen mustard/DNA alkylating chemotherapeutic, Chlorambucil, slows the proliferation rate but does not kill U2OS cells at any dose (non-lethal drug). For (A-C) drug GRADE for WT and KO cells calculated and shown below the respective plots.

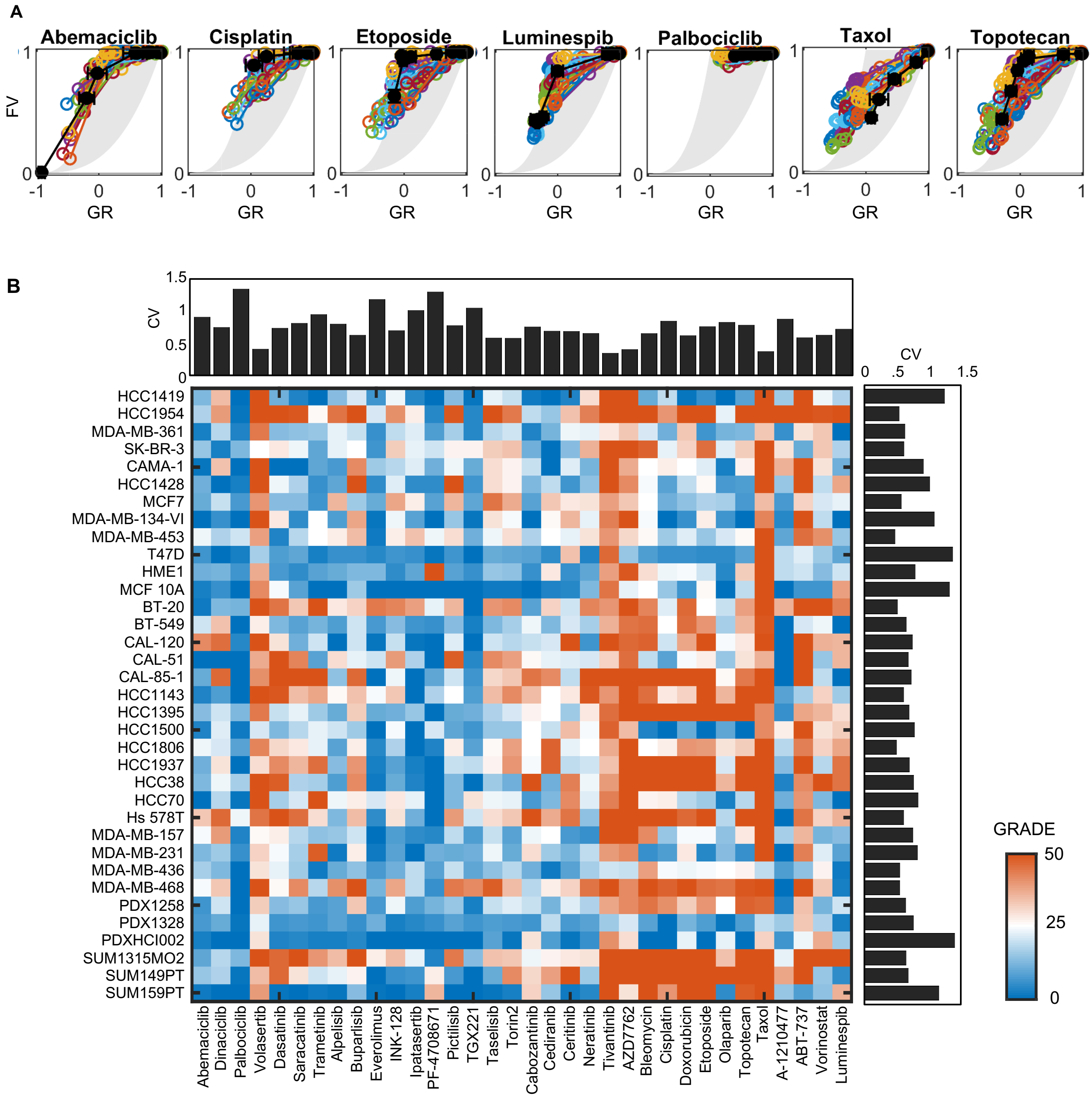

Figure S5: GRADEs for 34 drugs in 35 LINCS cell lines, Related to Figure 5. (A) GR/FV plots for 7 example drugs tested in Hafner et al. (2019), across 35 LINCS consortium cell lines. Mean GR/FV values for LINCS consortium cell lines are shown as colored dots with different doses of a drug connected by a line. U2OS is shown as a black dot and line. Error bars for U2OS are the standard deviation across 4 replicates. **(B)** Drug GRADEs for all 34 drugs tested in 35 LINCS cell lines. Drug GRADE for all drugs tested by in Hafner et al. (2019) across LINCS consortium cell lines. Heatmap colored according to GRADE. Bar plots show the coefficient of variation (CV) across all cell lines for a given drug, or across all drugs for a given cell line. The mean GRADE across all cells and drugs was 25.
