# Supplementary material for: Drug GRADE: An Integrated Analysis of Population Growth and Cell Death Reveals Drug-Specific and Cancer Subtype-Specific Response Profiles
Source: Cell Rep. Author manuscript; Available in PMC 2020 Jul 31. (PMC7394473; doi:10.1016/j.celrep.2020.107800)
Supplement: 5 [file NIHMS1607445-supplement-5.pdf]

Cell Reports

Article

Drug GRADE: An Integrated Analysis of Population Growth and Cell Death Reveals Drug-Specific and Cancer Subtype-Specific Response Profiles

Graphical Abstract

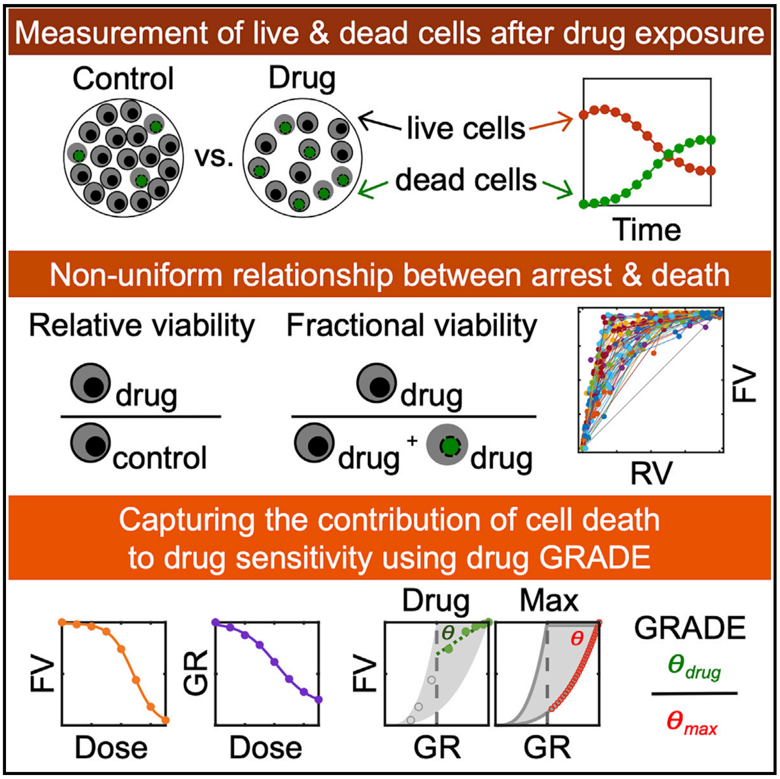

Highlights

- Relative viability and fractional viability score different aspects of drug action
- Drugs affect growth and cell death to different levels and with different timing
- GRADE measures the degree to which cell death contributes to a drug response
- GRADE captures subtype-dependent sensitivities that are missed using other methods

Authors

Hannah R. Schwartz, Ryan Richards, Rachel E. Fontana, Anna J. Joyce, Megan E. Honeywell, Michael J. Lee

Correspondence

michael.lee@umassmed.edu

In Brief

Anti-cancer drugs affect both the growth and survival of cancer cells. Commonly used measures of drug sensitivity do not distinguish between these two different outcomes. Schwartz et al. developed GRADE, a drug analysis method that reveals the proportional contributions of cell death versus growth inhibition for an observed drug response.

Schwartz et al., 2020, Cell Reports *31*, 107800
June 23, 2020 © 2020 The Author(s).
https://doi.org/10.1016/j.celrep.2020.107800

CellPress

Cell Reports

CellPress
OPEN ACCESS

Article

Drug GRADE: An Integrated Analysis of Population Growth and Cell Death Reveals Drug-Specific and Cancer Subtype-Specific Response Profiles

Hannah R. Schwartz,[1,3] Ryan Richards,[1,3] Rachel E. Fontana,[1] Anna J. Joyce,[1] Megan E. Honeywell,[1] and Michael J. Lee[1,2,4,*]
[1]Program in Systems Biology (PSB), University of Massachusetts Medical School, Worcester, MA, USA
[2]Program in Molecular Medicine (PMM), Department of Molecular, Cell, and Cancer Biology (MCCB), University of Massachusetts Medical School, Worcester, MA, USA
[3]These authors contributed equally
[4]Lead Contact
*Correspondence: michael.lee@umassmed.edu
https://doi.org/10.1016/j.celrep.2020.107800

SUMMARY

When evaluating anti-cancer drugs, two different measurements are used: relative viability, which scores an amalgam of proliferative arrest and cell death, and fractional viability, which specifically scores the degree of cell killing. We quantify relationships between drug-induced growth inhibition and cell death by counting live and dead cells using quantitative microscopy. We find that most drugs affect both proliferation and death, but in different proportions and with different relative timing. This causes a non-uniform relationship between relative and fractional response measurements. To unify these measurements, we created a data visualization and analysis platform called drug GRADE, which characterizes the degree to which death contributes to an observed drug response. GRADE captures drug- and genotype-specific responses, which are not captured using traditional pharmacometrics. This study highlights the idiosyncratic nature of drug-induced proliferative arrest and cell death. Furthermore, we provide a metric for quantitatively evaluating the relationship between these behaviors.

INTRODUCTION

Precise evaluation of the response of a cell to a drug is a critical step in pre-clinical drug development. Failures in this process have contributed to issues with irreproducibility of phenotypes across experimental platforms, spurious associations in precision medicine, and misannotated mechanisms of drug action (Bruno et al., 2017; Chopra et al., 2020; Hafner et al., 2019; Haibe-Kains et al., 2013). Recent studies continue to reveal that we generally do not know how drugs function, even for drugs that are well studied and precisely engineered (Lin et al., 2019). Traditional methods to evaluate a drug response have relied on pharmacological measures of the dose-response relationship of a drug, such as the half-maximal effective concentration (EC_{50}) or the half-maximal inhibitory concentration (IC_{50}). These features are important, but they reveal a biased and incomplete insight. Notably, measures of drug potency such as the EC_{50} or IC_{50} are poorly correlated with other important features, such as the maximum response to a drug (i.e., drug efficacy) (Fallahi-Sichani et al., 2013). Furthermore, measures of drug potency provide minimal insight into the mechanisms of drug action. In recent years, several drug-scoring algorithms have been developed to improve the evaluation of pharmacological dose responses, including approaches that facilitate an integrated evaluation of drug potency and efficacy (Fallahi-Sichani et al., 2013; Meyer et al., 2019). In addition, it has now been well demonstrated that differences in the proliferation rate between cell types were a confounding factor in most prior measurements of drug sensitivity (Hafner et al., 2016). Correcting for these artifactual differences in apparent drug sensitivity generates a more rational evaluation and has identified drug sensitivity-genotype relationships that are missed using traditional methods (Hafner et al., 2016; Harris et al., 2016).

One issue that has not been explored in detail is the underlying data itself. In nearly all cases, drug sensitivity is scored by comparing the relative number of live cells in the context of drug treatment to the number of live cells in a vehicle control condition. This metric is variably referred to as "relative viability," "percent survival," "percent viability," "drug sensitivity," "normalized cytotoxicity," and so forth (hereafter referred to as relative viability [RV]). RV is a convenient measure of drug response, and can be quantified using most commonly used population-based assays (e.g., MTT, CellTiter-Glo, Alamar blue, colony formation). Changes to RV can result from partial or complete arrest of cell proliferation, increased cell death, or both of these behaviors (Hafner et al., 2016). Because RV is determined entirely from live cells, this measure provides no insight into the number of dead cells, or more important, the relationship between proliferative arrest and cell death following the application of a drug. When using RV, it is generally unclear to

This is an open access article under the CC BY license (http://creativecommons.org/licenses/by/4.0/).

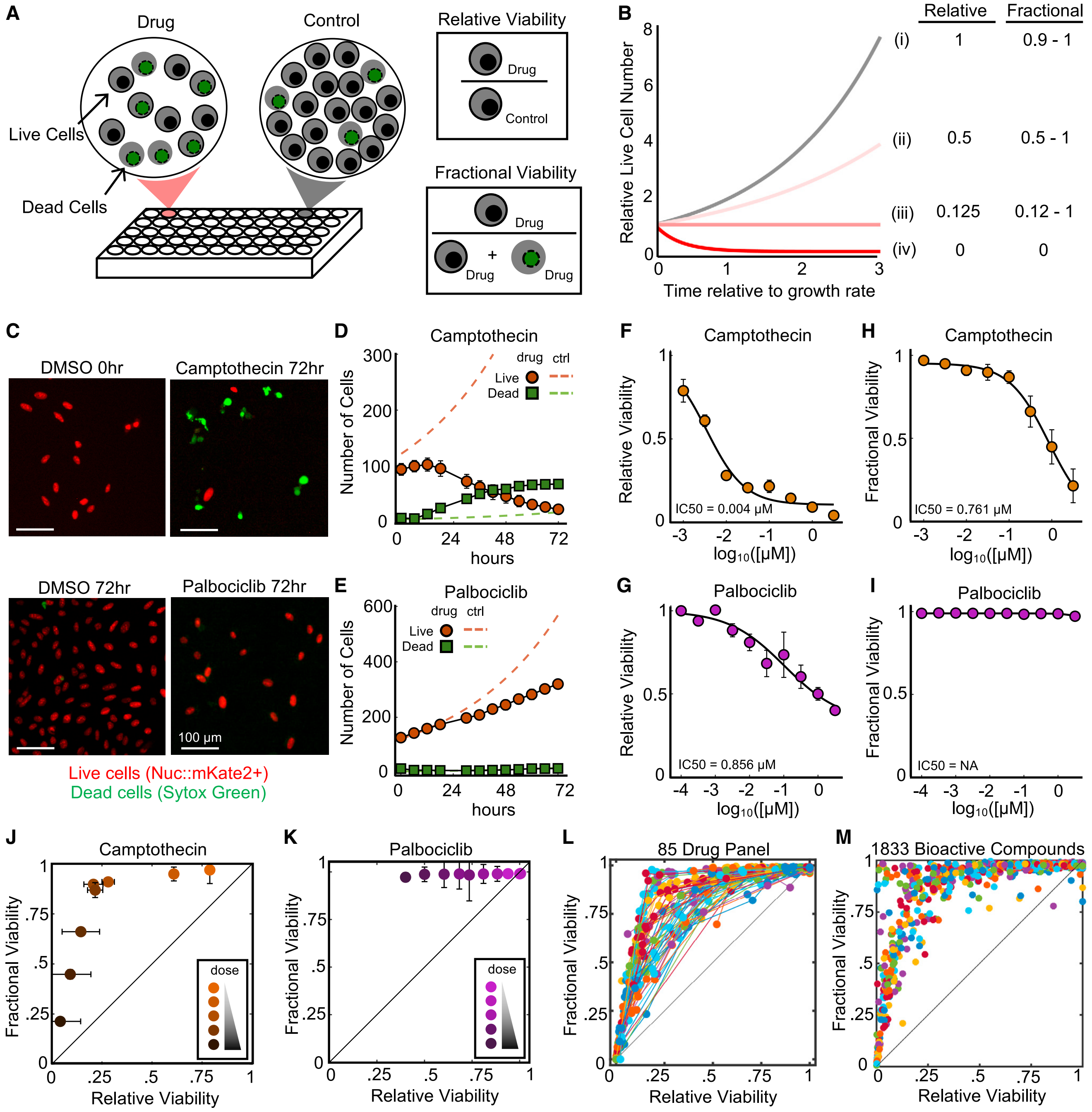

Figure 1. RV and FV Produce Largely Unrelated Insights into Drug Response

(A) Schematic defining common ways to quantify drug responses: fractional viability (FV) and relative viability (RV).

(B) Simulated data of drug response over time for (i) untreated, (ii and iii) partially cytostatic/cytotoxic, and (iv) fully cytotoxic conditions. RV and FV are values on a scale of 0–1 (RV = 1 means the population is 100% as large as the untreated; FV = 1 means the population is 100% alive).

(C–K) STACK assay to measure RV and FV. U2OS-Nuc::mKate2$^+$ cells treated with drug in the presence of SYTOX Green.

(C) Representative images from cells treated with either DMSO, 3.16 μM camptothecin, or 1 μM palbociclib. Scale bars in images represent 100 μm in length.

(D and E) Quantified live and dead cell counts over time for cells treated with camptothecin (D) or palbociclib (E), as in (C).

(F and G) RV dose-response functions for camptothecin (F) or palbociclib (G).

(H and I) FV dose-response functions for camptothecin (H) or palbociclib (I).

(J and K) RV versus FV at all doses for camptothecin (J) or palbociclib (K).

(legend continued on next page)

CellPress
OPEN ACCESS

what extent a cell population is undergoing proliferative arrest versus cell death at a given drug concentration (Figure 1A).

An alternative measure of drug sensitivity exists in which a drug response is quantified as the fractional proportion of live and dead cells in the drug-treated population (Figure 1A). This metric is variably called ‘‘lethal fraction’’ (or its inverse, ‘‘viable fraction’’), ‘‘percent of cells,’’ or ‘‘percent cell death’’ (hereafter referred to as fractional viability [FV]). In contrast to RV, FV provides direct insight into the degree of cell death within a population. In addition, FV calculations do not require comparison between treated and untreated groups, which minimizes issues associated with plating bias, a common issue in multi-well assays (Lachmann et al., 2016). In spite of these benefits, FV is less commonly used because this measure generally requires either extra measurements or the use of an experimental platform that provides single-cell data, such as in flow cytometry-based evaluation of apoptosis or quantitative microscopy (Albeck et al., 2008; Forcina et al., 2017).

Relative and fractional measures of drug response are often used interchangeably, in spite of the fact that these are clearly different metrics (Méry et al., 2017; Riss et al., 2019). In this study, we explored the relationship between these two common measures of drug sensitivity. We find that RV and FV score unique and largely unrelated properties of a drug response. RV accurately reports the cell population size, but not the degree of cell killing. Alternatively, FV exclusively reports drug-induced cell death, but does not provide any insight into the size of the surviving population. By directly comparing relative and fractional drug responses, we find that at any given dose, most drugs induce a coincident decrease in the cell proliferation rate and an increase in the cell death rate. Furthermore, when evaluating across a large panel of drugs, we find a non-uniform relationship between the inhibition of cell proliferation and the activation of cell death, spanning the entire continuum of possible behaviors. We find that the relative proportion of drug-induced proliferative inhibition versus cell death varies by drug, by dose, and by genotype. Furthermore, these features are not captured by traditional pharmacometrics such as the EC_{50} or IC_{50}. We developed a quantitative analysis platform called drug GRADE (growth rate-adjusted death) that captures the timing and relative magnitude of proliferative inhibition versus cell death. Evaluation of drug GRADE improves the ability to resolve cancer subtype-drug-response relationships. This study highlights the complex and non-uniform relationship between cell proliferation and cell death and provides an analytical framework for understanding these relationships.

RESULTS

RV and FV Produce Largely Unrelated Insights about Drug Response

In an effort to gain deeper insights into the mechanisms of action for common anti-cancer drugs, we began by exploring the relationship between two common measures of drug response: RV and FV (Figure 1A). A critical difference between these two measures is that RV is focused entirely on the live cell population across two conditions (drug treated and untreated), whereas FV includes both live and dead cells, but only in the drug-treated condition. In addition, because RV uses an untreated control as a reference point, this measure generally cannot distinguish between responses that are due to inhibiting proliferation versus those that are due to activating cell death (Hafner et al., 2016). Likewise, while decreased FV must require some degree of cell death, it is generally unclear whether death occurs in a proliferating, inhibited, or arrested population. Thus, while RV and FV should be correlated, if not identical, at extremely strong or weak response levels, the theoretical relationship between these numbers is unclear, particularly at intermediate levels of response (Figure 1B). We reasoned that exploring the relationship between RV and FV in detail could reveal hidden principles of drug sensitivity that are not captured using traditional measures. We evaluated drug responses in U2OS cells using the scalable time-lapse analysis of cell death kinetics (STACK) assay, a quantitative live-cell microscopy assay that measures both live and dead cells and has equal sensitivity in quantifying RV and FV (Forcina et al., 2017). We began by investigating RV and FV responses to two drugs: camptothecin, a topoisomerase I inhibitor and potent apoptotic agent, and palbociclib, a CDK4/6 inhibitor that primarily induces proliferative arrest without inducing any cell death (Hafner et al., 2019). As expected, camptothecin induced high levels of cell death, whereas palbociclib strongly inhibited the growth of the population without causing any cell death (Figures 1C–1E, S1A, and S1B).

To characterize the relationship between RV and FV responses, we profiled each drug using an eight-point half-log dose titration. From these data, we calculated both RV and FV metrics at the assay endpoint (Figures 1F–1I). A direct comparison of RV and FV for camptothecin revealed a discontinuous relationship featuring two clearly distinct dose-dependent behaviors (Figure 1J). In the first phase (low doses, which accounts for the majority of the RV scale), RV is strongly decreased in a dose-dependent manner while only modestly affecting FV. In the second phase (higher doses), FV decreases sharply while RV is only modestly affected (Figure 1J). These two phases reflect a decrease in proliferation rate with minimal cell killing at low doses, followed by an increase in death rate, which occurs at high doses and only in growth-arrested cells (Figure S1C). Alternatively, for palbociclib, which does not kill any cells, only the first of these two phases was observed (Figures 1K and S1C).

To determine whether biphasic response is a common behavior of many drugs or drug classes, we tested full dose-response profiles for a panel of 85 drugs, which target a variety of different proteins controlling cell proliferation and/or cell death (Table S1). For these drugs, the correlation between RV and FV

(L) RV versus FV at all doses for 85 cell death or growth-targeting drugs. Dots for a given drug represent the mean response at each tested dose. The dose titration for each drug is connected by a colored line.

(M) RV versus FV for 1,833 bioactive compounds, each tested at 5 μM.

For (D)–(K), data are means ± SDs of 4 replicates. Data in (M) are from Forcina et al. (2017).

See also Figure S1 and Table S1.

responses varied by drug, but they were generally not well correlated (Figures 1L and S1D). For some compounds, we observed a biphasic dose response similar to that of camptothecin, characterized by two linear but discontinuous phases, with death occurring only following full proliferative arrest. For most drugs, however, these two phases were more mixed, and doses were found in which the RV and FV values reported intermediate levels of proliferative inhibition and cell death. To supplement these data, we also reanalyzed a large publicly available dataset of 1,833 bioactive compounds that were previously tested using the STACK assay (Forcina et al., 2017). The overall profile of responses across these diverse compounds also highlights a spectrum of behaviors, rather than exclusively biphasic responses (Figure 1M). Thus, these data demonstrate that relative and fractional measures of drug response are not interchangeable and highlight the lack of a uniform relationship between FV and RV across drugs.

Relationships between RV and FV Vary Due to Idiosyncrasies in the Strength and Relative Timing of Drug-Induced Proliferative Inhibition versus Drug-Induced Cell Death

Overall, the IC_{50} doses computed using RV or FV (hereafter, RV_{50} and FV_{50}, respectively) were not well correlated, often differing by several orders of magnitude (Figures 1F–1I and 2A). The RV_{50} reports the dose at which the number of live cells following drug treatment is half as large as the untreated population, whereas the FV_{50} reports the dose at which a population is half alive and half dead (Figures S2A–S2C). Thus, these two values should be the same only in situations in which death occurs in the absence of any modulation to the proliferation rate of surviving cells (i.e., death in a population of cells that is otherwise proliferating at the normal rate). In theory, this could be achieved in several ways. For instance, drugs that induce death with a very fast onset time may kill cells before any observable changes in population size. The FV_{50} and RV_{50} values were very similar for particularly fast drugs, such as SGI-1027, a DNA methyltransferase 1 (DNMT1) inhibitor, and ABT-737, a BH3 mimetic (Figures 2B and 2C). To determine whether this was a general trend, we calculated the correlation between death onset time and the FV_{50}/RV_{50} ratio. We found a weak trend in which the FV_{50} and RV_{50} were more similar for drugs that had earlier onset times, but the overall correlation was modest, suggesting that death onset time alone was not a particularly good predictor of the FV/RV relationship ($r^2 = 0.3957$; Figure 2C).

In theory, other mechanisms exist, in addition to death onset time, that likely contribute to variations between FV and RV metrics. For instance, regardless of death onset time, FV and RV values would differ if a drug potently inhibited cell proliferation at low, non-killing doses, as we observed for drugs that induce biphasic responses such as camptothecin (Figure 1J). Likewise, even for drugs with very late death onset times, FV and RV values should still be similar if the onset time of proliferative inhibition was equally late. To identify such scenarios, we focused on drugs for which the death onset time was a poor predictor of the relationship between FV and RV, such as abemaciclib and entinostat.

FV_{50} and RV_{50} values for the CDK4/6 inhibitor abemaciclib were unusually varied, even for a drug with slow death onset time (Figures 2B and 2C). Consistent with our expectations, abemaciclib produced a distinctly biphasic dose response, characterized by strong growth inhibition at low non-lethal doses, and death only at high doses. (Figures 2D–2F). Furthermore, our comparisons of RV and FV values over time, rather than across doses, revealed that abemaciclib induces death only following a prolonged period of proliferative arrest (Figures S2D and S2E).

Alternatively, the histone deacetylase (HDAC) inhibitor entinostat induced death with a delayed onset time of ∼30 h after drug exposure, but nonetheless, FV and RV values were well correlated (Figures 2B and 2C). For this drug, kinetic analysis revealed that entinostat-treated cells proliferate at precisely the untreated rate for ∼30 h, such that the onset time of growth inhibition is equally delayed and similar to the onset time of cell death (Figures 2G–2I). Thus, these data highlight the lack of a singular "rule" describing the relationship between FV and RV values. The relationship between FV and RV depends on a combination of features, including the death onset time and whether cell death is occurring in a proliferating or an arrested population. These data also underscore the fact that common pharmacometrics derived from FV or RV fail to capture the relationship between drug-induced changes in proliferation versus cell death.

Integrative Analysis of Relative and Fractional Drug Responses Reveals a Continuum of Distinct Relationships between Growth Inhibition and Cell Death

RV measures different aspects of a drug response than FV. Because a simple rule could not be identified for predicting one from the other, we next asked what may be learned by quantitatively exploring the relationship between these metrics. We began by simulating RV and FV values for theoretical drug responses, using all possible combinations of fractional growth inhibition and fractional cell death in different proportions (Figures 3A and 3B). These simulations revealed an area of possible responses, with boundaries representing three distinct response scenarios: proliferative inhibition or arrest without any cell death (green line, top, Figure 3C), cell death within a population of normally proliferating cells (red line, right, Figure 3C), and a discontinuous biphasic response characterized by proliferative arrest at low doses, followed by cell death only within growth-arrested cells (blue line, top and left, Figure 3C).

The size and shape of this region varies dramatically, depending on the length of the assay and the proliferation rate assumed in the simulation. Thus, to stabilize these relationships, we also simulated drug responses using the normalized growth rate (GR) inhibition value. GR values are similar to RV in that both are derived from measurements of live cells in drug-treated and untreated conditions. A critical difference, however, is that the GR value scores a drug response based on a comparison of population GRs in the presence and absence of the drug, rather than scoring changes in population size as in RV (Hafner et al., 2016). Thus, GR corrects for artifactual differences in drug sensitivity that may be caused by differences in assay

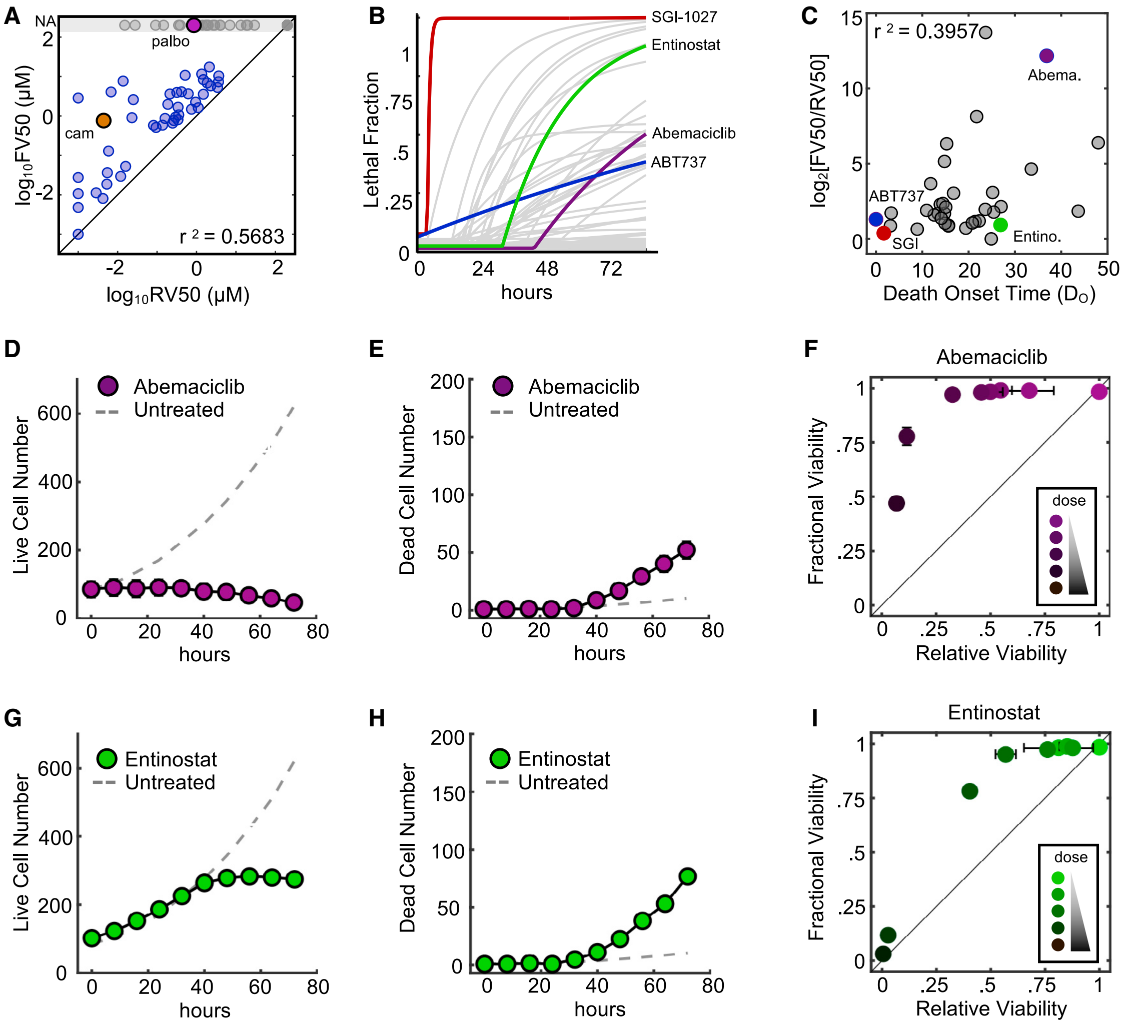

Figure 2. RV and FV Differ Due to Idiosyncrasies in the Strength and Relative Timing of Drug-Induced Proliferative Inhibition versus Cell Death

(A) Correlation between IC_{50} computed using RV (RV_{50}) or FV (FV_{50}). Pearson correlation coefficient shown.

(B) Death kinetics computed for 85 cell death and growth-inhibiting drugs. SGI-1027 (red), abemaciclib (purple), ABT-737 (blue), and entinostat (green) are highlighted.

(C) Correlation between death onset time (D_O) and the FV_{50}/RV_{50} ratio. Pearson correlation coefficient shown.

(D and E) Cell numbers over time for 10 μM abemaciclib. (D) Live cells. (E) Dead cells.

(F) Relationship between FV and RV for a dose range of abemaciclib (0–10 μM) at 72 h.

(G and H) Cell numbers over time for 3.16 μM entinostat. (G) Live cells. (H) Dead cells.

(I) Relationship between FV and RV for a dose range of entinostat (0–31.6 μM) at 72 h.

For (D)–(I), data are means ± SDs from 3 biological replicates.

See also Figure S2.

length between experiments or differences in proliferation rate between cell types. A comparison of simulated FV and GR values revealed a region of possible relationships defined by the same boundaries seen for FV versus RV comparisons (Figure 3D).

For both FV versus GR and FV versus RV comparisons, the area between the observed limits represents drug responses that feature both some growth inhibition and some cell death at varied proportions. From the simulated data, any data point within this bounded space can be attributed to a specific degree of fractional growth inhibition and cell death (region "b," Figures 3C and 3D; Table S2). The regions outside the bounded area represent responses that, while conceptually possible, are not observed in our simulated responses. Region "a" to the left of the bounded area would include drug responses in which the population size is decreased in excess of the measured number of dead cells (Figures 3C and 3D). This may be observed for some types of cell death, such as entosis (Overholtzer et al., 2007), and for technical reasons related to assay precision and/or the relative sensitivity of live cell and dead cell measurements. Region "c," to the right of the bounded area, includes

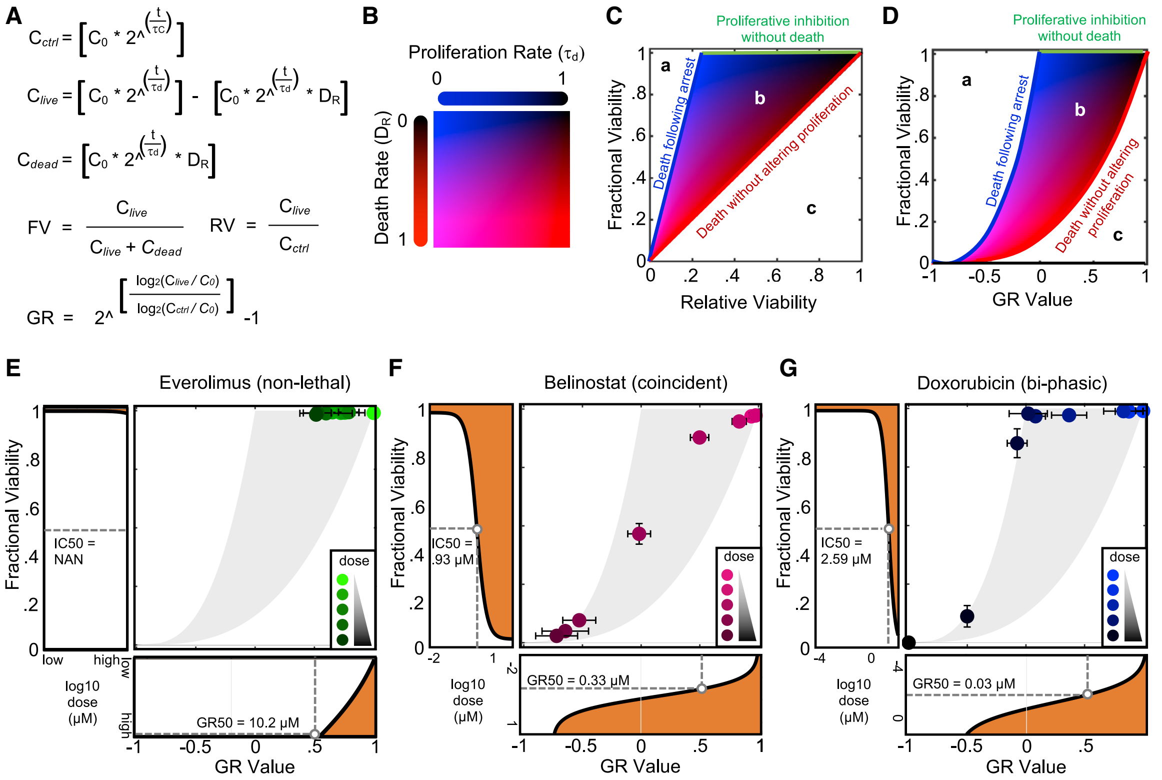

Figure 3. Integrative Analysis of Relative and Fractional Drug Responses Reveals a Continuum of Distinct Relationships between Drug-Induced Growth Arrest and Cell Death

(A–D) Simulations of all possible variations in drug-induced proliferation and cell death.

(A) Equations for live cells in control untreated condition (C_{ctrl}), live cells in drug-treated condition (C_{live}), dead cells in drug-treated condition (C_{dead}), FV, RV, and growth rate (GR) inhibition values. C_0, initial cell number; D_R, average death rate of drug-treated cells t, assay duration; t_c, GR of control (untreated cells); and t_d, GR of drug-treated cells. For this simulation, the death rate of control cells is presumed to be zero.

(B) Color map of parameter values. Red increases as death rate increases. Blue increases as GR decreases. The scale for death rate and GR are relative to the untreated GR.

(C) FV and RV calculated for full parameter space in (B).

(D) FV and GR calculated for full parameter space in (B).

(E–G) Examples of drug responses visualized through the integrated analysis of GR-FV. For each, the GR-FV plot is flanked by the FV dose-response profile (left) and the GR dose-response profile (bottom).

(E) GR-FV plot for everolimus, a drug that induces GR inhibition without cell death.

(F) GR-FV plot for belinostat, a drug that induces coincident GR inhibition with cell death.

(G) GR-FV plot for an example biphasic drug, doxorubicin.

Data in (E)–(G) are means ± SDs of 3 biological replicates.

See also Figure S3 and Table S2.

responses in which the degree of cell death is compensated for by a drug-induced increase in the proliferation rate (Figures 3C and 3D).

Although responses in regions “a” and “c” are possible in theory, these are never observed in our experimental data. For all 85 drugs profiled, the response data fell entirely within the bounds represented by region “b” (Table S2). Some drugs inhibited proliferation but were non-lethal at all tested doses (Figure 3E). Most drug responses, however, were characterized by GR and FV values that reveal partial growth suppression that occurs coincidentally with partial cell death, at different proportions for each drug (Figure 3F; Table S2). The responses of several drugs fell precisely at the top- and left-most boundaries, represented by biphasic dose-response profiles, including abemaciclib and most DNA-damaging chemotherapeutics (Figure 3G; Table S2).

These abrupt non-linear transitions likely capture critical changes in the drug mechanism of action that occur in a dose-dependent manner. For instance, it has been recently reported that abemaciclib-induced cell death occurs due to its off-target activity against CDK2, which is inhibited by abemaciclib exclusively at high doses (Hafner et al., 2019). Likewise, for DNA-damaging drugs, low levels of DNA damage are sufficient to induce cell-cycle arrest, but apoptotic cell death is only activated following higher levels of DNA damage (Figure S3). These dose-dependent transition points between proliferative inhibition and

cell death are clearly visible using a combined analysis of GR and FV (Figure 3G). This is notable, considering that these transition points are not generally observable in traditional analyses of dose-response data.

Drug GRADE Captures Distinct Drug Class-Specific Relationships between Drug-Induced Proliferative Arrest and Cell Death

By comparing the experimentally observed drug responses to our theoretical simulations, we calculated average proliferation rates and cell death rates for each drug, at each tested dose (Figures S4A–S4E; Table S2). These data further highlight that the degree to which a drug inhibits proliferation or activates cell death depends on the drug, but also strongly depends on the dose(s) of the drug tested (Figures S4D and S4E). Thus, we sought to create a summary metric, akin to the IC_{50}/EC_{50}, that captures the dose-dependent relationship between drug-induced cell death and proliferative arrest. As with the IC_{50} or EC_{50}, such a metric could be used to compare how responses differ by drug, by cancer subtype, or across different genotypes within a subtype.

Using the observed relationship between GR and FV values, we developed a metric that we call the drug GRADE (Figure 4A). The drug GRADE reports the proportion of an observed drug response that is due to cell death. We calculated the drug GRADE using the angle formed between a linear fit of the observed GR and FV data and a non-lethal drug response (Figures 4A and S4A, θ). This angle was calculated using a range of doses for which GR >0, as the relationship between FV and GR was approximately linear within this range. These data were further rescaled relative to the maximum angle possible within our simulated data, such that drug GRADE scales from 0 to 100, with 100 reporting that the observed response was entirely due to cell death and a GRADE of 0 reporting that the observed response was entirely due to inhibiting proliferation. Analysis of our kinetic data reveal that drug GRADE is reasonably stable for most drugs if measurements are taken between 48 and 72 h after drug addition (Figures S4F and S4G).

To explore the robustness of drug GRADE, we first evaluated whether targeted perturbations to cell death mechanisms would alter drug GRADE in a predictable manner. For instance, the inhibition of apoptosis using genetic knockout of BAX and BAK should inhibit cell death without compromising the drug-induced inhibition of cell proliferation. Furthermore, these changes should be specific to drugs that predominantly function by activating apoptotic cell death. To explore these predictions, we calculated drug GRADE for drugs that we recently characterized as inducing apoptotic death, non-apoptotic death, or non-lethal anti-proliferative responses (Richards et al., 2020). Consistent with expectations, ABT737, a BH3 mimetic and potent activator of apoptosis, had a very high drug GRADE, which was strongly diminished in the BAX-BAK double-knockout background (Figure S4H). In wild-type versus BAX-BAK double-knockout cells, drug GRADE was not significantly changed for JQ1, a Brd4 inhibitor that induces non-apoptotic death in U2OS cells; nor was drug GRADE altered for chlorambucil, a nitrogen mustard and DNA-alkylating agent that inhibited proliferation without activating cell death (Figures 4I and 4J). Thus, drug GRADE accurately captures the degree to which cell death contributes to an observed drug response.

Inspecting drug GRADE for the 85 drugs that we profiled revealed a continuous distribution of values, further demonstrating the unique drug-specific relationship between population growth inhibition and cell death (Figure 4B). Nonetheless, similarities were observed between drugs within a given class. For instance, DNA-damaging chemotherapeutics were enriched for very small drug GRADEs, indicating that for these drugs, the population reduction at IC_{50} doses is generally due to growth inhibition, rather than cell death (Figure 4C). Alternatively, microtubule toxins tended to have large drug GRADEs, indicating potent killing at IC_{50} doses (Figure 4C). Drug GRADE was not correlated with traditional pharmacometrics, such as the IC_{50}, EC_{50}, or E_{max} (Table S1). Thus, while traditional pharmacometrics report insights into drug affinity, potency, or efficacy, drug GRADE provides a unique insight into the mechanism of population reduction.

Drug GRADE Captures Subtype-Dependent Differences in Drug Sensitivity That Are Not Captured Using Traditional Pharmacometrics

Drug potency and drug efficacy are known to vary in a genotype- and cancer subtype-dependent manner. It was unclear whether drug GRADEs are stable features of a given drug or whether these would also vary for a given drug across cancer subtypes. To explore this question, we analyzed a publicly available dataset collected by the Library of Integrated Network-Based Cellular Signatures (LINCS) consortium, which contained 34 drugs tested across 35 breast cancer cell lines, with the data collected in a manner that would allow both GR and FV calculations (Hafner et al., 2019). For essentially all drugs, we found striking differences in drug GRADE across the cell lines (Figure S5). For instance, doxorubicin, a topoisomerase II inhibitor that is commonly used in the treatment of breast cancer, produced a biphasic dose response in U2OS cells, characterized by cell death only at high doses and only following full growth arrest (GRADE = 3.8; Figure 3G). In the LINCS breast cancer cell lines, however, doxorubicin GRADEs ranged from 1 to 73, revealing substantial variation in the degree of cell killing at IC_{50} doses (Figure 5A). Variation in drug GRADE was observed for all drugs, including targeted agents such as Torin 2 (Figures 4B, S5A, and S5B). Cell-cycle- and growth factor-targeted therapies were skewed toward smaller GRADEs, which is consistent with the notion that these drugs primarily induce growth inhibition, rather than cell death (Figure 5C). Cytotoxic chemotherapies, which can induce both growth inhibition and cell death, had a nearly random distribution of drug GRADEs across the cell lines studied (Figure 5C).

For cytotoxic chemotherapies, the observed variance in drug GRADE across cell lines may suggest that GRADE can capture genotype- or subtype-specific differences in drug response. An alternative explanation could be that the relationship between drug-induced growth arrest and drug-induced cell death is not determined by the drug, but instead is either stochastic or subject to strong environmental and/or context-dependent regulation. To distinguish between these possibilities, we investigated, for each cell line, the variation within GRADEs for drugs that

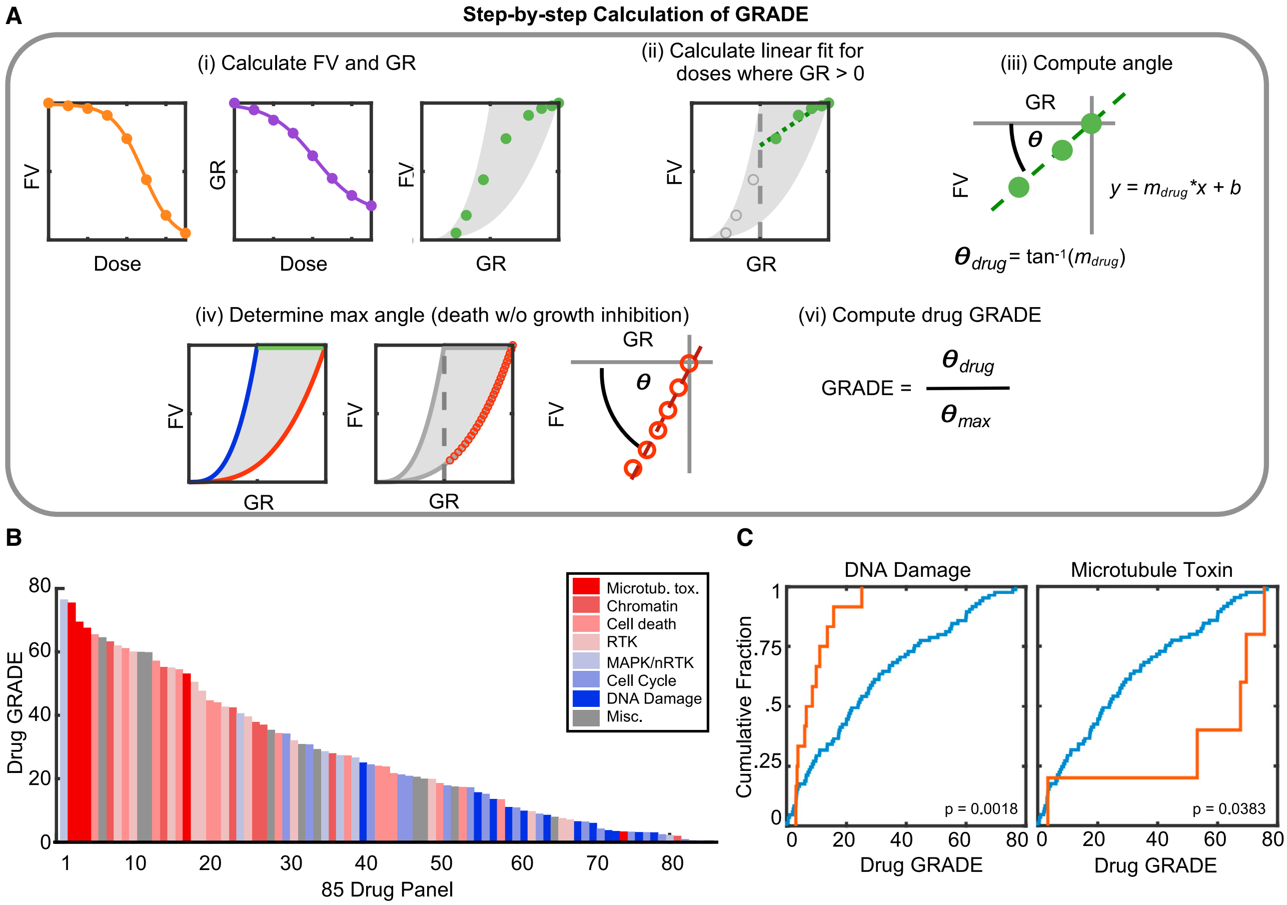

Figure 4. Drug GRADE Captures Distinct Drug Class-Specific Relationships between Drug-Induced Proliferative Arrest and Cell Death

(A) Step-by-step calculation of drug GRADE. See Experimental Model and Subject Details for a detailed description.

(B) Waterfall plot of GRADEs for 85 drugs tested.

(C) Cumulative distribution functions of drug GRADE for all 85 drugs (blue) or drugs in the listed class (orange). p values calculated using a 2-tailed Kolmogorov-Smirnov (KS) test.

See also Figure S4 and Table S1.

share a common mechanism of action. The LINCS dataset includes 6 different drugs that act by causing DNA damage, and 10 drugs annotated as phosphatidylinositol 3-kinase/mammalian target of rapamycin (PI3K/mTOR) inhibitors (Table S1). For any one of these drugs, significant variation was observed in drug GRADE across the LINCS cell lines (Figures 5A, 5B, and S5B). In contrast, within any given cell line, drugs of a shared class produced strikingly similar drug GRADEs (Figures 5D and S5B). Similar drug GRADEs were observed even for the DNA-damaging drug class, which included drugs that induce DNA damage using a variety of different molecular mechanisms, and through unrelated drug-binding targets. These data suggest that the variation observed for drug GRADE is related to the specific ways in which a given cell or cell type responds to a class of drugs.

The variations that are uncovered by drug GRADE reveal important differences in the underlying drug response. For instance, DNA-damaging agents resulted in biphasic dose responses and low drug GRADEs in T47D, a luminal estrogen receptor-positive (ER^+) breast cancer cell line (GRADE = 5.5; Figure 5D). In contrast, these drugs consistently resulted in coincident proliferative inhibition and cell death with high drug GRADEs in MDA-MB-468, a basal triple-negative breast cancer (TNBC) cell line (GRADE = 54.9; Figure 5D). This distinction reveals that the traditional IC_{50} (IC_{50} calculated from RV, RV_{50}) captures a partially growth-suppressing dose in T47D, but the same pharmacological value captures a potent killing dose in MDA-MB-468 (Figures 5E and 5F). Furthermore, while the IC_{50} values are similar and not statistically distinguishable for most DNA-damaging drugs in these two cell lines, they are generally lower in T47D when compared to MDA-MB-468, and generally lower in luminal cells when compared to TNBCs (Figures 5G and 5H). Thus, from the IC_{50} data alone, one may predict either equal chemosensitivity among breast cancer subclasses or that luminal breast cancer cells are more chemosensitive than TNBCs. These conclusions would be inconsistent with established clinical data, as TNBCs are well validated to be more chemosensitive than other breast cancer subtypes (Carey et al., 2007). While the

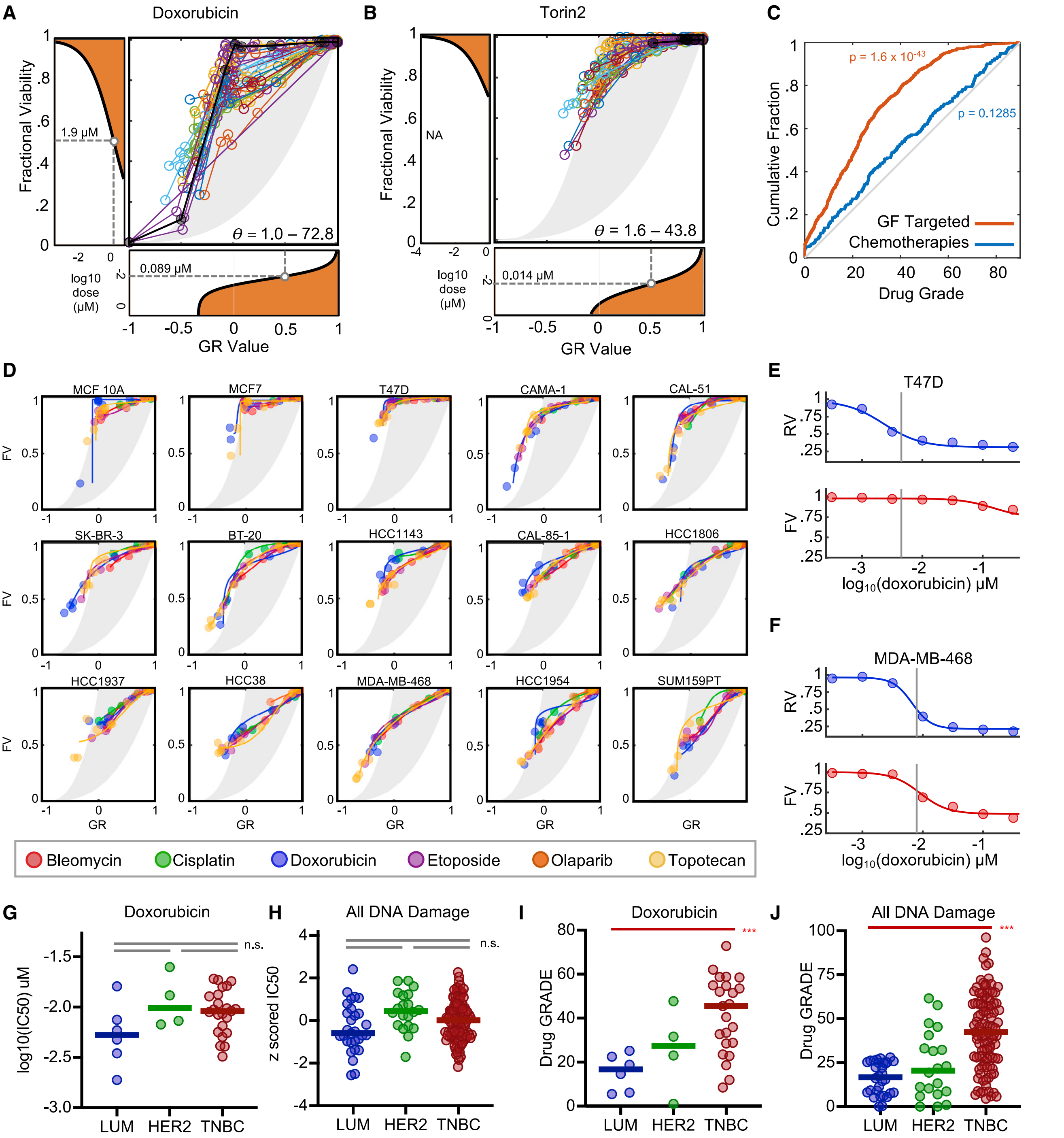

Figure 5. Drug GRADE Captures Subtype-Dependent Differences in Drug Sensitivity That Are Not Captured Using Traditional Pharmacometrics

(A and B) GR-FV plots for doxorubicin (A) or Torin 2 (B) for 35 cell lines from the LINCS dataset. U2OS data are shown in black for comparison. The range of GRADEs (θ) across all cell lines shown. GR and FV dose-response curves are for the mean responses across all cell lines.

(C) Cumulative distribution function of GRADEs for cytotoxic chemotherapies or growth factor-targeted therapies. The p value from the KS test is shown for deviation from random scores.

(D) GR-FV plots for 6 DNA-damaging drugs across 15 breast cancer cell lines from LINCS.

(E and F) RV and FV dose responses shown for doxorubicin in T47D (E) or MDA-MB-468 (F). Traditional IC_{50} (i.e., RV_{50}) highlighted with gray bar.

(legend continued on next page)

IC_{50} fails to capture subtype-specific differences in chemosensitivity, drug GRADE identifies significant differences between breast cancer subtypes. DNA-damaging drugs in TNBCs have significantly higher drug GRADEs than in other breast cancer subtypes, revealing that DNA-damaging chemotherapies induce greater levels of cell death in TNBCs than in other breast cancer subtypes (Figures 5I and 5J). These data highlight that drug GRADE captures critical differences in drug response that are not captured by traditional pharmacometrics.

DISCUSSION

Recent studies have revealed that differences in the population GR are a confounding factor in the measurement of the effectiveness of anti-cancer therapies (Hafner et al., 2016; Harris et al., 2016). These studies were a major step forward in analysis methods and have provided much needed clarity about mechanisms driving drug-induced changes in population size. The strategy we use here builds upon these prior works, and in fact, uses the GR value as one of the two key analysis features. A clear distinction, however, is that our approach integrates an independent measurement of dead cells and drug-induced FV. We find that the integrated analysis of population growth (through GR) and fractional killing (through FV) reveals drug- and cancer subtype-specific features of a drug response that are not captured using either of these values alone or when using any traditional pharmacometrics.

The most common measures of drug response are derived exclusively from measurements of live cells. Using these measurements to infer the degree of death requires some assumption to be made about the relationship between drug-induced proliferative inhibition and cell death. For instance, a common assumption is that cell death occurs only in growth-arrested cells. A central finding from our study is that the relationship between drug-induced proliferative inhibition and cell death varies substantially across drugs, and in a continuous manner. Also, for a given drug or drug class, drug GRADE varied substantially across cancer subtypes. Thus, in the absence of direct measurements of both FV- and RV-type responses, any assumption made regarding the relationship between the inhibition of proliferation and cell death is certain to be wrong in most situations.

Of note, the sign of the GR scale is generally interpreted as revealing the response phenotype, with positive GR values interpreted as partial inhibition of proliferation, whereas negative values are interpreted as cell death (more formally interpreted as a negative proliferation rate). Although it must be true that negative GR values report drug-induced cell death, notably, positive GR values do not necessarily report the lack of cell death. This was clearly demonstrated in theory in the original description of the GR value (Hafner et al., 2016), and our analysis reveals that for most drugs, significant levels of death are observed in the positive portion of the GR scale. These phenotypes generally resulted from intermediate levels of cell death occurring within a population of cells that continue to proliferate. Thus, while the GR value unambiguously reports the net population GR in a manner that distinguishes between an increasing and a decreasing population size, whether a drug induces significant killing requires additional measurements. The strategy we describe in this study clarifies this issue, and our data show that GR and FV values provide complementary insights into the nature of a drug response.

Using the complementary insights generated by GR and FV measures, we found that TNBCs respond to low doses of DNA-damaging chemotherapies by activating cell death, whereas luminal breast cancers respond by halting cell proliferation. TNBCs are known to have higher levels of chemosensitivity than other breast cancer subtypes. In some cases, these differences are related to deficiencies in DNA repair, but in most cases, it remains unclear which factors account for the varied levels of sensitivity to DNA-damaging chemotherapies (Heijink et al., 2019). Drug GRADE analysis may be a valuable tool in identifying molecular or genomic features that contribute to chemosensitivity, particularly since differences in chemosensitivity between TNBC and other breast cancer subtypes were not observed in traditional measurements of drug response.

One limitation of the analysis method we propose is that it cannot be used in conjunction with many common drug-response assays that exclusively measure live cells (e.g., CellTiter-Glo, MTT, Alamar Blue, colony formation). Our approach should be amenable to any assay that develops single-cell data for live and dead cells, such as flow cytometry, histology, or the microscopy-based STACK analysis used in this study. In addition, we recently developed a high-throughput fluorescent plate reader-based strategy for inferring live cell counts using only a direct measurement of dead cells (Richards et al., 2020). When combined with the drug GRADE analysis from this study, these high-throughput methods, which also rely on SYTOX fluorescence, are particularly useful for comparing across various types of apoptotic and/or non-apoptotic death. SYTOX fluorescence is specific to cell death but largely agnostic to the mechanism by which cells die. Thus, if only live or dead cells can be counted, our data suggest that the measurement of dead cells would be preferable, as live cells can be accurately inferred using modest experimental and computational adjustments (Richards et al., 2020).

Drug-response assays are common to many sectors of biomedical research, and a common practice is to summarize drug responses using measures such as the IC_{50}, EC_{50}, or E_{max}. These metrics are used to compare across drugs or to compare drug responses across biological scenarios. In many situations, such as oncology, a critical question generally remains unanswered by these metrics: does the drug actively kill cells or just inhibit cell proliferation? This is an important

(G and H) Traditional IC_{50}s for doxorubicin (G) or all DNA-damaging drugs (H) across 36 cell lines. Data are separated by breast cancer subtype: luminal (LUM), HER2 overexpressing (HER2), or triple-negative (TNBC).

(I and J) Drug GRADE for doxorubicin (I) or all DNA-damaging drugs (J) across 36 cell lines. Data are separated as in (G) and (H).

For (G)–(J), t test p values are shown for a comparison of TNBC to LUM. All other comparisons are not significant. ***$p < 0.05$; n.s. = not significant.

See also Figure S5.

distinction. Inhibiting proliferation is not likely to result in a durable response in the absence of other interventions, such as surgery or additional therapies, particularly when considering the rapid clearance of most chemotherapeutics due to drug metabolism and excretion. In current approaches, a common strategy to determine if an observed response is due to cell death or growth inhibition is to use RV to characterize drug potency and/or efficacy. These measures are then complemented with a more specific measure of cell death to determine whether the observed response was caused by growth arrest or cell death. Our study reveals a flaw in this line of thinking, that the response was necessarily "either/or" and not "both." We find that most drugs achieve their effects through some combination of population growth inhibition and cell death, but the relative proportions of these effects vary by drug, by dose, and across different cancer subtypes. Clarifying these relationships should improve our ability to accurately evaluate drug responses and how these responses vary across drugs or across biological contexts.

STAR★METHODS

Detailed methods are provided in the online version of this paper and include the following:

- KEY RESOURCES TABLE
- RESOURCE AVAILABILITY
  - Lead Contact
  - Materials Availability
  - Data and Code Availability
- EXPERIMENTAL MODEL AND SUBJECT DETAILS
  - Cell lines and culture conditions
  - Chemicals and reagents
- METHOD DETAILS
  - Cell Seeding and Drug Addition
  - Live Cell Image Acquisition
  - Flow Cytometry Analysis of Drug Response
- QUANTIFICATION AND STATISTICAL ANALYSIS
  - Data analysis and statistics
  - Quantitative Image Analysis
  - Flow Cytometry Analysis
  - Calculation of Drug GRADE
  - Use and interpretation of drug GRADE
  - Modeling Growth Curves
  - Drug Dose Response Analysis

SUPPLEMENTAL INFORMATION

Supplemental Information can be found online at https://doi.org/10.1016/j.celrep.2020.107800.

ACKNOWLEDGMENTS

We thank current and past members of the UMassMed PSB community for their helpful comments and critiques during the design and execution of this study. In addition, we thank M. Hafner, S. Peyton, J. Pritchard, and M. Walhout for their helpful comments during the preparation of this manuscript. This work was supported by the National Institute of General Medical Sciences of the National Institutes of Health (NIH) (R01GM127559, to M.J.L.); the American Cancer Society (RSG-17-011-01, to M.J.L.); and a NIH/National Cancer Institute (NCI) training grant (Translational Cancer Biology Training Grant T32-CA130807, to R.R.).

AUTHOR CONTRIBUTIONS

This project was conceived by H.R.S., R.R., and M.J.L. The drug-response data were collected by H.R.S., R.R., R.E.F., A.J.J., and M.J.L. The data analysis was performed by H.R.S., R.R., M.E.H., and M.J.L. The manuscript was written and edited by H.R.S., R.R., and M.J.L.

DECLARATION OF INTERESTS

The authors declare no competing interests.

Received: March 3, 2020
Revised: May 1, 2020
Accepted: June 1, 2020
Published: June 23, 2020

REFERENCES

Albeck, J.G., Burke, J.M., Aldridge, B.B., Zhang, M., Lauffenburger, D.A., and Sorger, P.K. (2008). Quantitative analysis of pathways controlling extrinsic apoptosis in single cells. Mol. Cell *30*, 11–25.

Bruno, P.M., Liu, Y., Park, G.Y., Murai, J., Koch, C.E., Eisen, T.J., Pritchard, J.R., Pommier, Y., Lippard, S.J., and Hemann, M.T. (2017). A subset of platinum-containing chemotherapeutic agents kills cells by inducing ribosome biogenesis stress. Nat. Med. *23*, 461–471.

Carey, L.A., Dees, E.C., Sawyer, L., Gatti, L., Moore, D.T., Collichio, F., Ollila, D.W., Sartor, C.I., Graham, M.L., and Perou, C.M. (2007). The triple negative paradox: primary tumor chemosensitivity of breast cancer subtypes. Clin. Cancer Res. *13*, 2329–2334.

Chopra, S.S., Jenney, A., Palmer, A., Niepel, M., Chung, M., Mills, C., Sivakumaren, S.C., Liu, Q., Chen, J.-Y., Yapp, C., et al. (2020). Torin2 Exploits Replication and Checkpoint Vulnerabilities to Cause Death of PI3K-Activated Triple-Negative Breast Cancer Cells. Cell Syst. *10*, 66–81.e11.

Fallahi-Sichani, M., Honarnejad, S., Heiser, L.M., Gray, J.W., and Sorger, P.K. (2013). Metrics other than potency reveal systematic variation in responses to cancer drugs. Nat. Chem. Biol. *9*, 708–714.

Forcina, G.C., Conlon, M., Wells, A., Cao, J.Y., and Dixon, S.J. (2017). Systematic Quantification of Population Cell Death Kinetics in Mammalian Cells. Cell Syst. *4*, 600–610.e6.

Hafner, M., Niepel, M., Chung, M., and Sorger, P.K. (2016). Growth rate inhibition metrics correct for confounders in measuring sensitivity to cancer drugs. Nat. Methods *13*, 521–527.

Hafner, M., Mills, C.E., Subramanian, K., Chen, C., Chung, M., Boswell, S.A., Everley, R.A., Liu, C., Walmsley, C.S., Juric, D., and Sorger, P.K. (2019). Multiomics Profiling Establishes the Polypharmacology of FDA-Approved CDK4/6 Inhibitors and the Potential for Differential Clinical Activity. Cell Chem. Biol. *26*, 1067–1080.e8.

Haibe-Kains, B., El-Hachem, N., Birkbak, N.J., Jin, A.C., Beck, A.H., Aerts, H.J.W.L., and Quackenbush, J. (2013). Inconsistency in large pharmacogenomic studies. Nature *504*, 389–393.

Harris, L.A., Frick, P.L., Garbett, S.P., Hardeman, K.N., Paudel, B.B., Lopez, C.F., Quaranta, V., and Tyson, D.R. (2016). An unbiased metric of antiproliferative drug effect in vitro. Nat. Methods *13*, 497–500.

Heijink, A.M., Everts, M., Honeywell, M.E., Richards, R., Kok, Y.P., de Vries, E.G.E., Lee, M.J., and van Vugt, M.A.T.M. (2019). Modeling of Cisplatin-Induced Signaling Dynamics in Triple-Negative Breast Cancer Cells Reveals Mediators of Sensitivity. Cell Rep. *28*, 2345–2357.e5.

Lachmann, A., Giorgi, F.M., Alvarez, M.J., and Califano, A. (2016). Detection and removal of spatial bias in multiwell assays. Bioinformatics *32*, 1959–1965.

Lin, A., Giuliano, C.J., Palladino, A., John, K.M., Abramowicz, C., Yuan, M.L., Sausville, E.L., Lukow, D.A., Liu, L., Chait, A.R., et al. (2019). Off-target toxicity

is a common mechanism of action of cancer drugs undergoing clinical trials. Sci. Transl. Med. *11*, eaaw8412.

Méry, B., Guy, J.-B., Vallard, A., Espenel, S., Ardail, D., Rodriguez-Lafrasse, C., Rancoule, C., and Magné, N. (2017). In Vitro Cell Death Determination for Drug Discovery: A Landscape Review of Real Issues. J. Cell Death *10*, 1179670717691251.

Meyer, C.T., Wooten, D.J., Paudel, B.B., Bauer, J., Hardeman, K.N., Westover, D., Lovly, C.M., Harris, L.A., Tyson, D.R., and Quaranta, V. (2019). Quantifying Drug Combination Synergy along Potency and Efficacy Axes. Cell Syst. *8*, 97–108.e16.

Overholtzer, M., Mailleux, A.A., Mouneimne, G., Normand, G., Schnitt, S.J., King, R.W., Cibas, E.S., and Brugge, J.S. (2007). A nonapoptotic cell death process, entosis, that occurs by cell-in-cell invasion. Cell *131*, 966–979.

Richards, R., Schwartz, H.R., Honeywell, M.E., Stewart, M.S., Cruz-Gordillo, P., Joyce, A.J., Landry, B.D., and Lee, M.J. (2020). Drug antagonism and single-agent dominance result from differences in death kinetics. Nat. Chem. Biol. https://doi.org/10.1038/s41589-020-0510-4.

Riss, T., Niles, A., Moravec, R., Karassina, N., and Vidugiriene, J. (2019). Cytotoxicity Assays: In Vitro Methods to Measure Dead Cells. In The Assay Guidance Manual, N.P. Coussens and G.S. Sittampalam, eds. (Eli Lilly & Company and the National Center for Advancing Translational Sciences).

CellPress
OPEN ACCESS

STAR★METHODS

KEY RESOURCES TABLE

REAGENT or RESOURCE	SOURCE	IDENTIFIER
Antibodies		
Purified Rabbit Anti-Active Caspase-3	BD Biosciences	559565; RRID: AB_397274
Phospho-Histone H2A.X (Ser139) (20E3) Rabbit mAb	Cell Signaling Technology	9718; RRID: AB_2118009
Goat anti-Rabbit IgG(H+L) Cross-Absorbed Secondary Antibody, Alexa Fluor 488	ThermoFisher Scientific	A-11008; RRID: AB_143165
Chemicals, Peptides, and Recombinant Proteins		
A23187	ApexBio Technology	Cat#B6646
ABT-263 (Navitoclax)	ApexBio Technology	Cat#A3007
ABT-737	ApexBio Technology	Cat#A8193
Artesunate	ApexBio Technology	Cat#B3662
Axitinib (AG 013736)	ApexBio Technology	Cat#A8370
AZD2461	ApexBio Technology	Cat#A4164
Belinostat (PXD101)	ApexBio Technology	Cat#A4096
BI 2536	ApexBio Technology	Cat#A3965
Bleomycin Sulfate	ApexBio Technology	Cat#A8331
Bortezomib (PS-341)	ApexBio Technology	Cat#A2614
Bromodomain Inhibitor, (+)-JQ1	ApexBio Technology	Cat#A1910
BX795	ApexBio Technology	Cat#A8222
Cediranib (AZD217)	ApexBio Technology	Cat#A1882
Chlorambucil	ApexBio Technology	Cat#B3716
Dacarbazine	ApexBio Technology	Cat#A2197
Docetaxel	ApexBio Technology	Cat#A4394
Entinostat (MS-275,SNDX-275)	ApexBio Technology	Cat#A8171
Everolimus (RAD001)	ApexBio Technology	Cat#A8169
Flubendazole	ApexBio Technology	Cat#B1759
Flumequine	ApexBio Technology	Cat#B2292
Foretinib	ApexBio Technology	Cat#A2974
GSK J1	ApexBio Technology	Cat#A4191
Honokiol	ApexBio Technology	Cat#N1672
JNJ-26854165 (Serdemetan)	ApexBio Technology	Cat#A4204
MG-132	ApexBio Technology	Cat#A2585
MK1775	ApexBio Technology	Cat#A5755
Niclosamide	ApexBio Technology	Cat#B2283
Nigericin sodium salt	ApexBio Technology	Cat#B7644
Nilotinib	ApexBio Technology	Cat#A8232
Oubain	ApexBio Technology	Cat#B2270
Paclitaxel (Taxol)	ApexBio Technology	Cat#A4393
Panobinostat (LBH589)	ApexBio Technology	Cat#A8178
Pazopanib Hydrochloride	ApexBio Technology	Cat#A8347
PD 0332991 (Palbociclib) HCl	ApexBio Technology	Cat#A8316
RITA (NSC 652287)	ApexBio Technology	Cat#A4202
RSL3	ApexBio Technology	Cat#B6095
Sabutoclax	ApexBio Technology	Cat#A4199
Salinomycin	ApexBio Technology	Cat#A3785
SB743921 HCl	ApexBio Technology	Cat#B1590

(*Continued on next page*)

Continued

REAGENT or RESOURCE	SOURCE	IDENTIFIER
SGI-1027	ApexBio Technology	Cat#B1622
TAE684 (NVP-TAE684)	ApexBio Technology	Cat#A8251
Temozolomide	ApexBio Technology	Cat#B1399
TH287	ApexBio Technology	Cat#B5849
Tivozanib (AV-951)	ApexBio Technology	Cat#A2251
Topotecan HCl	ApexBio Technology	Cat#B2296
Torin 1	ApexBio Technology	Cat#A8312
Torin 2	ApexBio Technology	Cat#B1640
Triptolide	ApexBio Technology	Cat#A3891
TW-37	ApexBio Technology	Cat#A4234
Vinblastine sulfate	ApexBio Technology	Cat#A3920
Vincristine	ApexBio Technology	Cat#A1765
Vorinostat	ApexBio Technology	Cat#A4084
YM-155 HCl	ApexBio Technology	Cat#A3947
Erastin2	Cayman Chemical	Cat#27087
Erlotinib	LC Laboratories	Cat#E-4007
Valinomycin	Millipore-sigma	Cat#V0627
A-1210477	Selleck Chemicals	Cat#S7790
Abemaciclib	Selleck Chemicals	Cat#S5716
Alpelisib	Selleck Chemicals	Cat#S2814
AZD7762	Selleck Chemicals	Cat#S1532
Bibf-1120 (Nintedanib)	Selleck Chemicals	Cat#S1010
Buparlisib (BKM120, NVP-BKM120)	Selleck Chemicals	Cat#S2247
Cabozantinib (XL184, BMS-907351)	Selleck Chemicals	Cat#S1119
Camptothecin	Selleck Chemicals	Cat#S1288
Ceritinib (LDK378)	Selleck Chemicals	Cat#S7083
Cisplatin	Selleck Chemicals	Cat#S1166
Dasatinib	Selleck Chemicals	Cat#S1021
Dinaciclib (SCH727965)	Selleck Chemicals	Cat#S2768
Erastin	Selleck Chemicals	Cat#S7242
Etoposide	Selleck Chemicals	Cat#S1225
INK-128 (Sapanisertib, MLN0128,TAK-228)	Selleck Chemicals	Cat#S2811
Ipatasertib (GDC-0068)	Selleck Chemicals	Cat#S2808
Luminespib (AUY-922, NVP-AUY922)	Selleck Chemicals	Cat#S1069
Neratinib	Selleck Chemicals	Cat#S2150
Olaparib (AZD2281, Ku-0059436)	Selleck Chemicals	Cat#S1060
PF-4708671	Selleck Chemicals	Cat#S2163
Pictilisib (GDC-0941)	Selleck Chemicals	Cat#S1065
Saracatinib (AZD0530)	Selleck Chemicals	Cat#S1006
SMER 28	Selleck Chemicals	Cat#S8240
Taselisib (GDC 0032)	Selleck Chemicals	Cat#S7103
TGX221	Selleck Chemicals	Cat#S1169
Tivantinib	Selleck Chemicals	Cat#S2753
Trametinib (GSK1120212)	Selleck Chemicals	Cat#S2673
Volasertib	Selleck Chemicals	Cat#S2235
Doxorubicin hydrochloride	Sigma Aldrich	Cat#D1515-10MG
Sytox Green Nucleic Acid Stain	ThermoFisher Scientific	Cat#S7020

(*Continued on next page*)

CellPress
OPEN ACCESS

Continued

REAGENT or RESOURCE	SOURCE	IDENTIFIER
Deposited Data		
GRADE plot function	This paper	https://github.com/MJLee-Lab/GRADE
Pharmacological response data for 85 drugs studied	This paper	Table S1
Proliferation and death rates for 85 drugs at each dose	This paper	Table S2
Experimental Models: Cell Lines		
U-2-OS::Nuc	Richards et al., 2020	https://pubmed.ncbi.nlm.nih.gov/32251407
Software and Algorithms		
Incucyte S3	Essen Biologics	2019B
MATLAB	MathWorks	R2019a
Prism	GraphPad	8.3.1

RESOURCE AVAILABILITY

Lead Contact

Further information and requests for resources and reagents should be directed to and will be fulfilled by the Lead Contact, Michael Lee (michael.lee@umassmed.edu).

Materials Availability

This study did not generate new unique reagents.

Data and Code Availability

Source data collected for a panel of 85 drugs at varied doses in U2OS cells are included in Tables S1 and S2. Images and raw cell counts from images have not been deposited in a public repository due to file size but will be made available upon request. Custom MATLAB code for computing drug GRADE and generating FV/GR plots are included in Data S1 and on GitHub (https://github.com/MJLee-Lab/GRADE). Custom MATLAB scripts for image analysis or curve fitting will be made available upon request.

EXPERIMENTAL MODEL AND SUBJECT DETAILS

Cell lines and culture conditions

This study uses U2OS cells, which were generated from a female with osteosarcoma. U2OS cells were obtained from ATCC, and authenticated by STR profiling. Additional analysis was also performed on the LINCS breast cancer cell lines, a panel of 35 cell lines derived from female donors with various subtypes of breast cancer (Hafner et al., 2019). mKate2 expressing U2OS cells were generated as previously described (Richards et al., 2020). Cells were grown in Dulbecco's modified eagles medium (DMEM) (Cat# MT10017CV, Fisher Scientific) supplemented with 10% fetal bovine serum (Cat# SH30910.03, Lot# AYC161519, ThermoFisher Scientific), 2 mM L-glutamine (Cat# 02500cl, Fisher Scientific), and penicillin/streptomycin (Cat# 30-002-Cl, Corning). Cell lines were cultured in incubators at 37C with 5% CO2. For passaging, cells were rinsed with PBS, dissociated with 0.25% trypsin (Cat# 15090-046, Life Technologies), quenched with complete DMEM, and counted using a hemocytometer. Cells were seeded for experiments as described in the Method Details section.

Chemicals and reagents

Sytox Green Nucleic Acid Stain (Cat#: S7020) was purchases from ThermoFisher Scientific (Waltham, MA). A23187 (Cat# B6646), ABT-263 (Navitoclax) (Cat# A3007), ABT-737 (Cat# A8193), Artesunate (Cat# B3662), Axitinib (AG 013736) (Cat# A8370), AZD2461 (Cat# A4164), Belinostat (PXD101) (Cat# A4096), BI 2536 (Cat# A3965), Bleomycin Sulfate (Cat# A8331), Bortezomib (PS-341) (Cat# A2614), Bromodomain Inhibitor, (+)-JQ1 (Cat# A1910), BX795 (Cat# A8222), Cediranib (AZD217) (Cat# A1882), Chlorambucil (Cat# B3716), Dacarbazine (Cat# A2197), Docetaxel (Cat# A4394), Entinostat (MS-275,SNDX-275) (Cat# A8171), Everolimus (RAD001) (Cat# A8169), Flubendazole (Cat# B1759), Flumequine (Cat# B2292), Foretinib (Cat# A2974), GSK J1 (Cat# A4191), Honokiol (Cat# N1672), JNJ-26854165 (Serdemetan) (Cat# A4204), MG-132 (Cat# A2585), MK1775 (Cat# A5755), Niclosamide (Cat# B2283), Nigericin sodium salt (Cat# B7644), Nilotinib (Cat# A8232), Oubain (Cat# B2270), Paclitaxel (Taxol) (Cat# A4393), Panobinostat (LBH589) (Cat# A8178), Pazopanib Hydrochloride (Cat# A8347), PD 0332991 (Palbociclib) HCl (Cat# A8316), RITA (NSC 652287) (Cat# A4202), RSL3 (Cat# B6095), Sabutoclax (Cat# A4199), Salinomycin (Cat# A3785), SB743921 HCl (Cat# B1590), SGI-1027 (Cat# B1622), TAE684 (NVP-TAE684) (Cat# A8251), Temozolomide (Cat# B1399), TH287 (Cat# B5849), Tivozanib (AV-951) (Cat# A2251), Topotecan HCl (Cat# B2296), Torin 1 (Cat# A8312), Torin 2 (Cat# B1640), Triptolide (Cat# A3891), TW-37 (Cat# A4234), Vinblastine sulfate (Cat# A3920), Vincristine (Cat# A1765), Vorinostat (Cat# A4084), and YM-155 HCl (Cat# A3947) were purchased from ApexBio

Technology (Houston, TX). Erastin2 (Cat# 27087) was purchased from Cayman Chemicals (Ann Arbor, MI). Erlotinib (Cat# E-4007) was purchased from LC Laboratories (Woburn, MA). Valinomycin (Cat# V0627) was purchased from MilliporeSigma (Burlington, MA). A-1210477 (Cat# S7790), Abemaciclib (Cat# S5716), Alpelisib (Cat# S2814), AZD7762 (Cat# S1532), Bibf-1120 (Nintedanib) (Cat# S1010), Buparlisib (BKM120, NVP-BKM120) (Cat# S2247), Cabozantinib (XL184, BMS-907351) (Cat# S1119), Camptothecin (Cat# S1288), Ceritinib (LDK378) (Cat# S7083), Cisplatin (Cat# S1166), Dasatinib (Cat# S1021), Dinaciclib (SCH727965) (Cat# S2768), Erastin (Cat# S7242), Etoposide (Cat# S1225), INK-128 (Sapanisertib, MLN0128,TAK-228) (Cat# S2811), Ipatasertib (GDC-0068) (Cat# S2808), Luminespib (AUY-922, NVP-AUY922) (Cat# S1069), Neratinib (Cat# S2150), Olaparib (AZD2281, Ku-0059436) (Cat# S1060), PF-4708671 (Cat# S2163), Pictilisib (GDC-0941) (Cat# S1065), Saracatinib (AZD0530) (Cat# S1006), SMER 28 (Cat# S8240), Taselisib (GDC 0032) (Cat# S7103), TGX221 (Cat# S1169), Tivantinib (Cat# S2753), Trametinib (GSK1120212) (Cat# S2673), and Volasertib (Cat# S2235) was purchased from Selleck Chemicals (Houston, TX). Doxorubicin HCl (Cat# D1515-10MG) was purchased from Sigma-Aldrich (St. Louis, MO).

METHOD DETAILS

Cell Seeding and Drug Addition

U2OS::mkate2+ cells were grown in 10cm dishes (Cat # FB012924, Fisher Scientific). Prior to drug treatment (" Day −1"), cells were trypsinized, counted using a hemocytometer. Experiments were performed in 96-well black-sided optical bottom plates (Cat # 3904, Corning), with cells seeded at a concentration of 2500 cells per 90 μL of media. Following overnight incubation at 37°C with 5% CO_2, drugs were added in growth media containing 500 nM SYTOX Green (10 μL volume; final concentration of 50 nM SYTOX in the well). Eight- or ten-point half log or full log dilutions for each compound were prepared in 96-well U-bottom storage plates (Cat #: 07-200-95, Corning) at 10x of their final concentration. Images was collected using the STACK assay (Forcina et al., 2017). Briefly, images were acquired using the IncuCyte S3 (Essen Biosciences) with settings for the green channel: ex: 460 ± 20; em: 524 ± 20; acquisition time: 300ms; and red channel: ex:585 ± 20; em: 635 ± 70; acquisition time: 400ms. Data were acquired either every 6-8 hours for 72 hours, or only at 72 hours when kinetic analysis was not needed.

Throughout the study, experiments were performed in biological triplicate. All data were used without omission of any replicates. Sample size was based on effect sizes and error observed in our prior study using similar methods (Richards et al., 2020). When multi-well plates (e.g., 96-well plates) were used, conditions were not randomized, but analysis did evaluate biases associated with plating location, which were found to be minimal. Edge wells were not used due to compromised proliferation rates.

Live Cell Image Acquisition

Images was collected using the STACK assay detailed in Forcina et al. (2017). Images were acquired using the IncuCyte S3 microscope (Essen Biosciences; 1408x1040 pixels, at 1.24 μm/pixel). Acquisition settings for the green channel were ex: 460 ± 20, em: 524 ± 20, acquisition time: 300ms; and red channel were ex:585 ± 20, em: 635 ± 70, acquisition time: 400ms. Imaging was performed using a 10x objective. For all experiments, on Day 0 just prior to drug addition, images were taken of a control plate treated with growth media containing 500 nM SYTOX Green as detailed above. For kinetic analysis, images were acquired every 6-8 hours for every well of each plate for 72 hours. For experiments where kinetic analysis was not used images were collected only at the 72 hour end point.

For some experiments that did not require kinetic analysis, images were acquired using an EVOS FL Auto 2 automated microscope (ThermoFisher Scientific). Images were acquired using a 10x objective (EVOS 10x objective, Cat #: AMEP4681). Sytox images were acquired using a GFP filter cube (EVOS LED Cube, GFP, Cat #: AMEP4651, ex: 470/22, em: 525/50, acquisition time: 13.5ms) Mkate2+ images were acquired using a TexasRed filter cube (EVOS LED Cube TxRed, Cat #: AMEP4655, ex: 585/29, em: 628/32, acquisition time: 642.0ms).

Flow Cytometry Analysis of Drug Response

Cells were seeded in 6-well dishes at 200,000 cells per well and allowed to attach overnight prior to drug treatment. At selected time points cells were washed in PBS, trypsinized, and fixed in 70% ethanol overnight at −20 C, permeabilized with 0.25% Triton X-100 for 20 minutes at 4 C and blocked with 1% BSA. For analysis of drug-induced apoptosis, cells were stained with antibodies against cleaved caspase-3 for 8 hours (1:250 dilution; CAT# 559565, BD Biosciences). For analysis of drug-induced DNA double stranded breaks, cells were stained with antibodies against phospho-histone H2A.X for 8 hours (1:200 dilution, CAT# 9718, Cell Signaling Technologies). Following washing with PBS, cells were incubated with a goat-anti-rabbit secondary antibody conjugated to Alexa488 (1:250 dilution; CAT# A-11008, ThermoFisher Scientific). Flow cytometry data were collected on a LSR II flow cytometer running FACS DIVA software.

QUANTIFICATION AND STATISTICAL ANALYSIS

Data analysis and statistics

Statistical details can be found in the figure legends, including statistical tests used, exact value and definition of n, definition of center, and dispersion and precision measures. Death kinetic rates (D_O and D_R) were determined using MATLAB, as described

previously (Richards et al., 2020). Statistical enrichments were determined in MATLAB using built-in functions 'kstest2' or 'fishertest' as indicated in the figure legends.

Quantitative Image Analysis

All images collected using the IncuCyte S3 system were analyzed using the IncuCyte Software (Essen Biosciences). Cell counting parameters were empirically determined using untreated cells and a subset of cytotoxic compounds. Analysis settings for SYTOX Green+ objects were: Top-Hat segmentation; Radius (μm) between 50 and 100; Threshold(GCU) between 5 and 10; Edge split on; Edge sensitivity between −25 and −45; Filter area min between 20 and 55; Filter area max between 2600 and 3000; Max eccentricity between 0.90 and 0.95. Analysis settings for mkate2+ objects were: Top-hat segmentation, Radius(μm) between 100 and 110; Threshold(GCU) between 0.8 and 1; Edge split on; Edge sensitivity between −45 and −35; Filter area(μm^2) max between 100 and 110; Filter area(μm^2) max between 2600 and 3000. The counts per well for the Sytox+ and mkate2+ objects were exported to excel and loaded into MATLAB for further analysis. For some experiments that did not require kinetic analysis, images were acquired using an EVOS FL Auto automated microscope. For images obtained using the EVOS microscope, the images were analyzed using custom MATLAB scripts, available upon request.

Flow Cytometry Analysis

Flow cytometry data were analyzed using FlowJo (v. 10.5.3). For gating cells of interest, FSC/SSC were used to identify cells, and FL2-A versus FL2-H was used to identify single cells. Cell cycle stage was quantified from the PI intensity using the FlowJo Cell Cycle analysis built-in function, using the Dean-Jett-Fox algorithm. To quantify apoptotic cells and/or cells with DNA damage for each cell cycle stage, area gates were used based on the negative control untreated samples.

Calculation of Drug GRADE

See also Figures 4A and S4 for a step-by-step guide for calculation of drug GRADE. Live cell and dead cell data generated from microscopy were used to calculate "fractional viability" (live cells divided by total cells; FV). In this study, FV data were not normalized (i.e., raw data were used), as the baseline cell death observed in U2OS cells in the absence of any drug was very low. In cell lines which have high basal levels of death, FV values will be much lower than 1 even without any drug exposure. In these cases, GRADE could be calculated from FV values normalized relative to the basal death rate. Growth rate inhibition metrics (GR) were calculated as described (Hafner et al., 2016). To calculate drug GRADE, we focused on all doses of a given drug that are less than or equal to the GR50 dose. Our experimental and simulated data show that the relationship between FV and GR is roughly linear for GR values between 0 – 1. Thus, for these doses the relationship between GR and FV were fit to a linear function. For most studies, the majority of the RV scale is captured within the GR 0 −1 range, including the IC_{50} dose. The GR50 is highly correlated with the traditional IC_{50} (i.e., IC_{50} from an RV dose response curve), so focusing on the positive portion of the GR scale means that drug GRADE will capture the degree to which cell death contributes to responses observed at the IC_{50} dose. Drug GRADE was determined using the following equation:

$$GRADE = \frac{tan^{-1}(m_{drug})}{tan^{-1}(m_{max})}$$

where tan^{-1} is the inverse tangent ('atan' function in MATLAB), m_{drug} is the slope of the linear fit relationship between FV and GR for doses of GR where GR is greater than or equal to zero, and m_{max} is the maximum slope observed over the same range of GR values, given the assumption that the observed response was entirely due to cell death, without any drug-induced slowing of cell proliferation. The maximum possible slope was determined from simulated experiments as described in Figure 3. Thus, drug GRADE reports as a percentage the contribution of cell death to the observed response at IC_{50} dose. A custom function for computing drug GRADE is available on GitHub (https://github.com/MJLee-Lab/GRADE) and included as Data S1.

Use and interpretation of drug GRADE

Drug GRADE can be calculated using data derived from any experimental platform that provides independent single cell measurements of live and dead cells, including flow cytometry, microscopy, or a SYTOX based plate reader assay (Richards et al., 2020). If the measurement of cell death is agnostic to the mechanism of killing, GRADE can be used to compare drugs that kill by any mechanism. GRADE values vary from 0 – 100 and report the degree to which cell death contributes to a drug response. For instance, a GRADE of 50 means that 50% of the observed response was due to cell death, with the remainder caused by proliferative arrest. Drug GRADE can be calculated from the relationship between FV and RV, or FV and GR. If making comparisons between cell types, we recommend using FV and GR, as the GR measurement corrects for artifactual differences in drug response related to differences in assay length or proliferation rate between cell types. For calculation of drug GRADE key considerations include the doses of drug tested and the time point(s) analyzed. Regarding doses, stable GRADEs require multiple data points for which GR is between 0 – 1. Ideally, the majority of this range should also be captured within the doses tested. GRADE can be calculated from essentially any dose series (2-fold, half-log, log dilution, etc.), given that multiple doses produce responses within the GR 0 – 1 range. For drugs that are essentially non-functional (GR and FV values > 0.9 at all doses), drug GRADEs are noisy and should not be calculated. These

limitations/considerations are similar for drug GRADE and for more traditional pharmaco-metrics such as the EC_{50}. Regarding time of analysis, because FV measures drug-induced cell death, it is critical that measurements be made after the onset time of cell death. Death onset times vary by drug, and by dose. For drugs in this study, GRADEs change over time but are stable by approximately 48 hours after drug addition.

For some particularly efficacious or toxic drugs, GR values shift at consecutive doses from GR $\sim$1 (no response) to GR < 0 (strong killing resulting in a negative population size). In these situations, only 1 or 0 data points would fall within the desired window for calculation of GRADE. Drug GRADE should not be calculated from single doses; however, single dose measurements of FV and GR can be used to compute average death rates and average proliferation rates. At any given dose, the average proliferation rate and death rate of the population can be determined based on the location of the data in the FV/GR plot. An example is shown in Figures S4D and S4E. Similar to drug GRADE, these values report the relative contribution of cell death and inhibition of proliferation to the observed response at a given dose. For these data, the death rate and proliferation rate are reported relative to the proliferation rate of untreated cells (i.e., 0.05 means 5% of the untreated proliferation rate).

Modeling Growth Curves

The experimental growth curves in this paper were fit using MATLAB's fit function with the equation: $y = a\Delta 2^{bx}$, where x is time of analysis, y was the number of live cells at time x, b is the proliferation rate in population doublings per hour, and *a* is a free coefficient. The *a* and *b* parameters were fit using nonlinear least-squares. Upper and lower bounds of *a* parameter were constrained using the min and max of y, respectively. Upper and lower bounds of the *b* parameter were constrained as 1/100 and 1/10, respectively.

Drug Dose Response Analysis

All dose response functions for relative viability and fractional viability were modeled using a 4-parameter logistic regression model:

$$\left(y = a + \frac{d-a}{1+10^{(x-b)c}}\right)$$

where *x* is the log10 transformed drug dose, *y* is the observed response in RV or FV, *a* is the E_{inf}, *b* the log10 transformed EC_{50}, *c* the Hill coefficient, and *d* the maximum *y* value. Fitting error was minimized using the nonlinear least-squares method. The lower limits for *a*, *b*, *c*, and *d* were 0, min(x)-2, 0.1, and 0; upper limits for *a*, *b*, *c*, and *d* were 1, max(x)+2, 5, and 1; start points for fitting *a*, *b*, *c*, and *d* were 0.5, (median(x)), 1, and 1. GR values were generated as described (Hafner et al., 2016). The GR dose response data was modeled using a 4-parameter logistic regression model as detailed above, with the exception that the lower limit of *a* was -1.

Cell Reports, Volume *31*

Supplemental Information

Drug GRADE: An Integrated Analysis of Population Growth and Cell Death Reveals Drug-Specific and Cancer Subtype-Specific Response Profiles

Hannah R. Schwartz, Ryan Richards, Rachel E. Fontana, Anna J. Joyce, Megan E. Honeywell, and Michael J. Lee

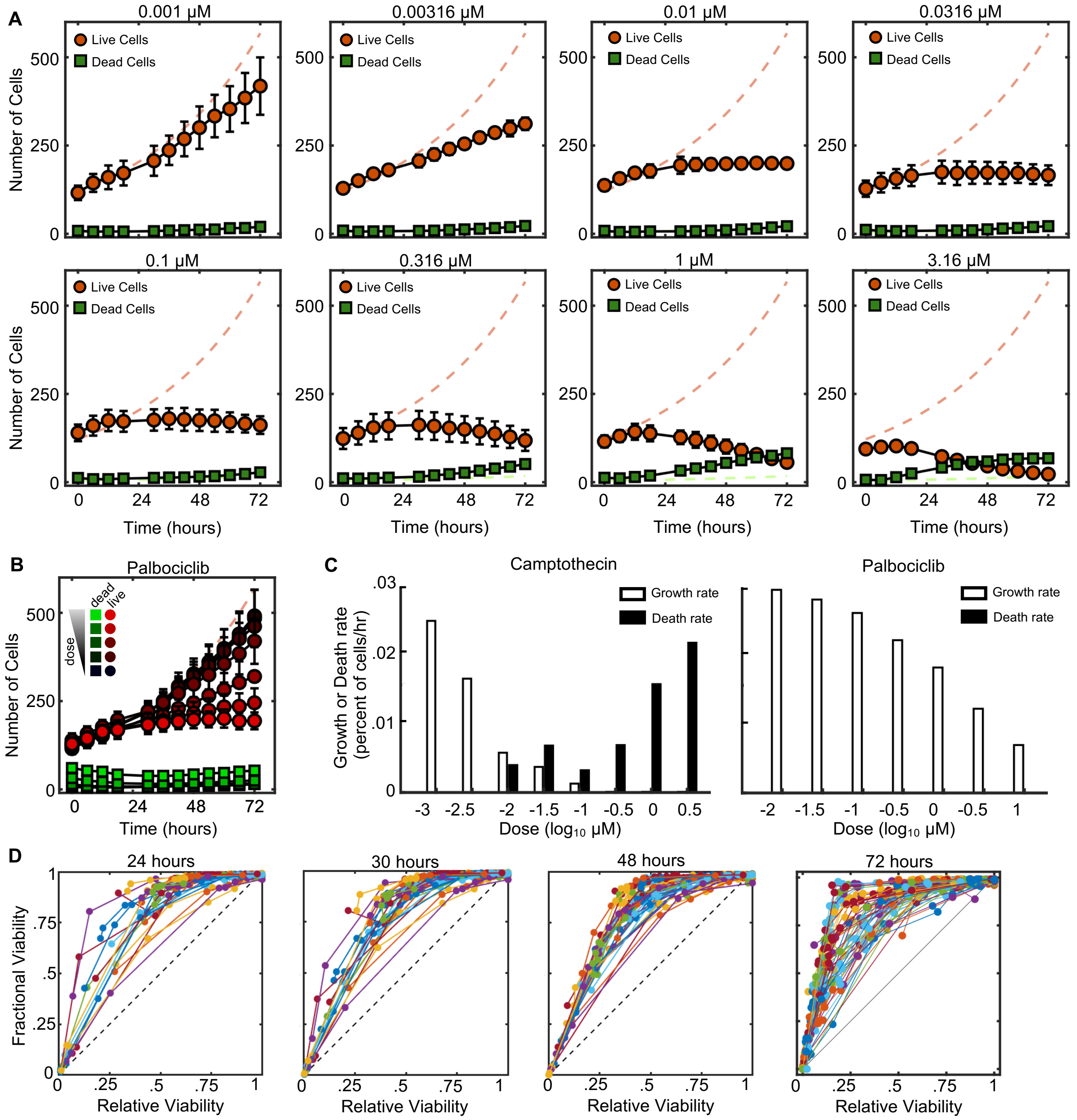

Figure S1: The relationship between relative and fractional responses differ by drug, Related to Figure 1. (A) The number of live and dead cells present over a 72-hour time course at different concentrations of Camptothecin, a bi-phasic drug. Dashed red and green are live and dead cell numbers for control untreated cells, respectively. **(B)** The number of live and dead cells present over a 72-hour time course at different concentrations of Palbociclib (10, 3.16, 1, 0.316, 0.1, 0.0316, or 0.01 µM). For (A) and (B) data are mean +/- s.d. of four replicates. **(C)** The growth rate and death rate calculated using an exponential growth model or lag-exponential death model, respectively, for each dose of Camptothecin and Palbociclib shown in panels (A) and (B). **(D)** RV and FV measured for a panel of 85 drugs at different time points as indicated. See also Table S1.

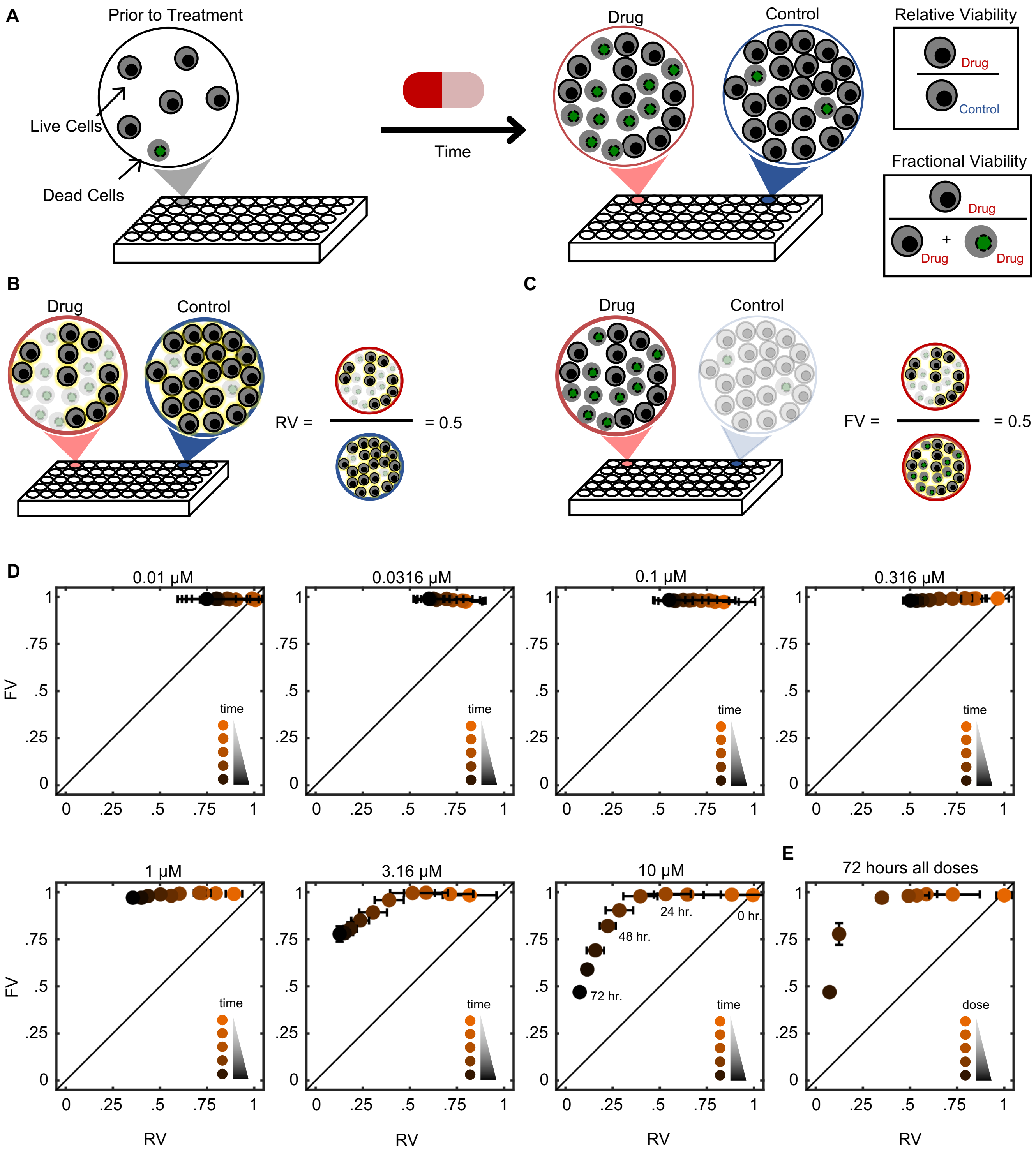

Figure S2: Relationship between RV50 and FV50, Related to Figure 2. (A) Schematic of a typical drug response assay in multi-well plates. Relative Viability (RV) and Fractional Viability (FV) are two common measures of response. **(B)** RV calculated for the example shown in (A). The example shown represents the IC50 of the RV measure (i.e. RV50), defined as the dose at which the observed number of live cells after drug exposure is half the size of the untreated population. **(C)** FV calculated for the example shown in (A). The example shown represents the IC50 of the FV measure (i.e. FV50), defined as the dose at which the population is half alive and half dead. Note, although both values in (B) and (C) are 0.5, these are computed from different comparisons and are defined differently. **(D)** RV/FV plots for each dose of Abemaciclib tested over a 72-hour time course using the STACK assay. Images were acquired every 8 hours. **(E)** RV versus FV plot for varied doses 72-hours after drug exposure. For panels (D) and E) data are mean +/- s.d. of two replicates.

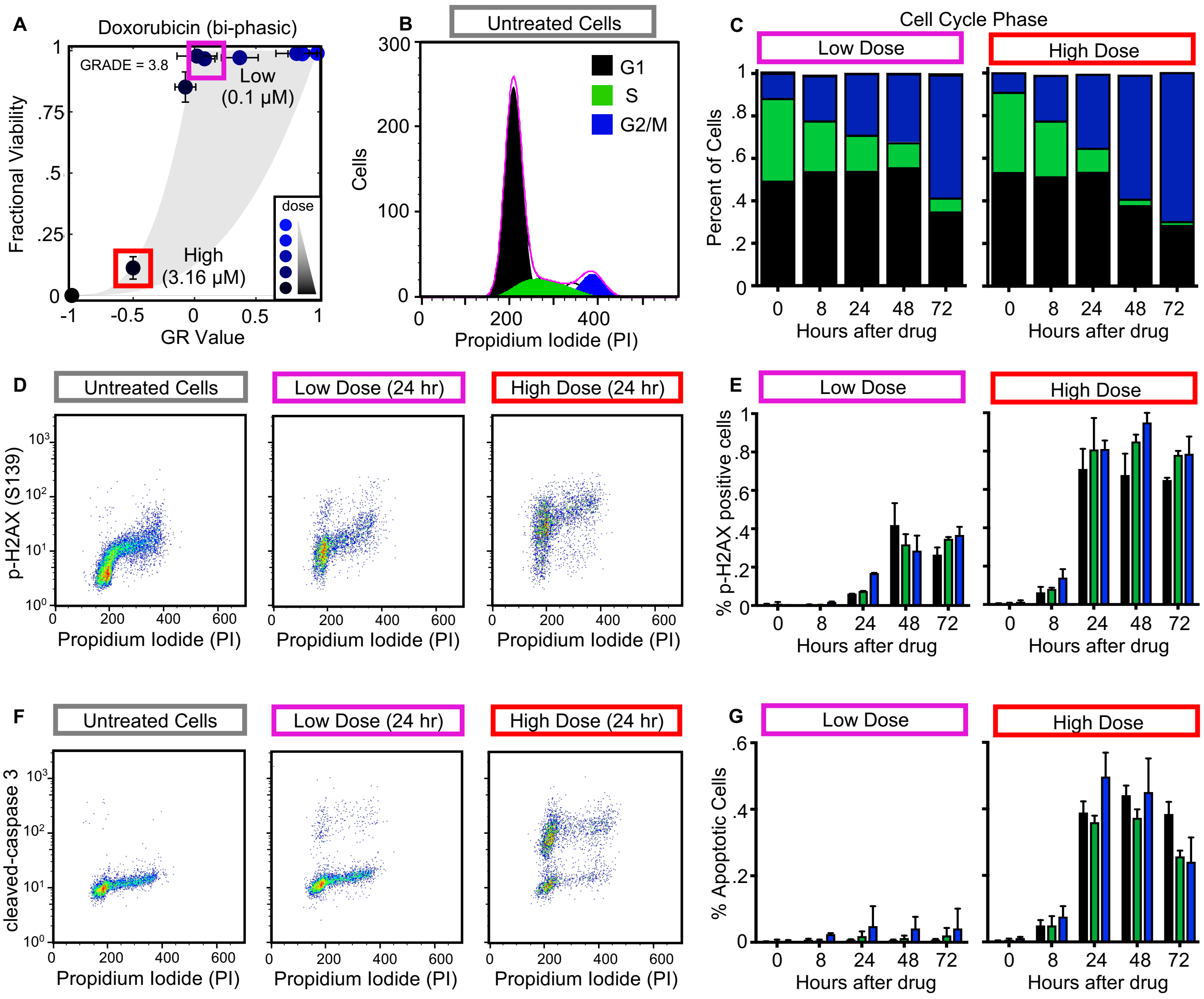

Figure S3: DNA damaging agents induce a bi-phasic drug response, Related to Figure 3. (A) Drug GR/FV plot for doxorubicin (DOX). Two doses highlighted: 0.1 µM (Low), which induces growth arrest, and 3.16 µM (High), which induces cell death but only following growth arrest. Data are mean +/- s.d. of 3 replicates. **(B-C)** Cell cycle analysis by flow cytometry using propidium iodide staining (PI). (B) Representative data for untreated cells. Cell cycle phase as determined by Dean-Jett-Fox algorithm shown. Data are representative of 3 biological replicates. (C) Cell cycle analysis for cells treated with Low or High dose DOX for indicated times. Colors are as in panel (B). **(D-E)** Analysis of DNA damage levels by flow cytometry. DNA double stranded breaks tracked using phosphorylation of H2AX (p-H2AX) in combination with cell cycle analysis using PI. (D) Representative data for untreated cells or cells exposed to Low or High dose DOX for 24 hours. Data are representative of 3 biological replicates. (E) H2AX phosphorylation kinetics for Low and High dose DOX. % H2AX positive cells determine by flow cytometry as in panel (D). Data are separated by cell cycle phase as in panels (B-C). Data are mean +/- s.d. of 3 biological replicates. **(F-G)** Flow cytometry-based analysis of apoptotic death in combination with cell cycle analysis using PI. (F) Representative data for untreated cells or cells exposed to Low or High dose DOX for 24 hours. Data are representative of 3 biological replicates. (G) Percentage of cleaved-caspase-3 positive cells measured using flow cytometry. Data are separated by cell cycle phase as in panels (B-C). Data are mean +/- s.d. of 3 biological replicates.

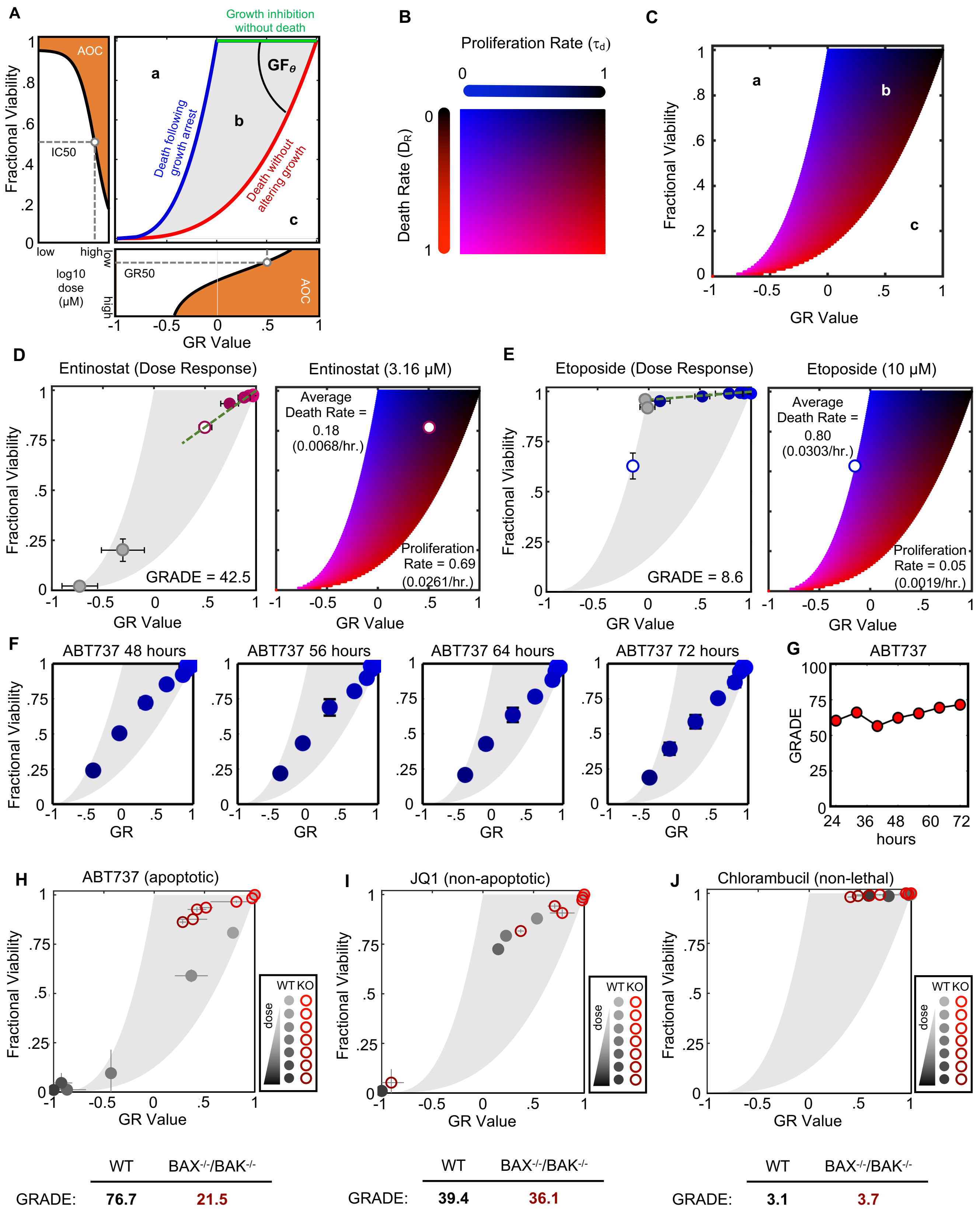

Figure S4: Stability and robustness of drug GRADE, Related to Figure 4. (A) Schematic of the GR/FV plot with reference limits shown for growth inhibition without death (green), death only following growth arrest (blue), and death without altering growth rate (red). Shaded region 'b' represents intermediate states in which a drug induces some growth inhibition and some death. **(B-C)** Simulated drug responses using all possible variations of drug-induced growth rate and death rates. **Figure legend continued on next page.**

Figure S4: Stability and robustness of drug GRADE, Related to Figure 4 (continued from previous page). (B) Color map of parameter values. Red increases as death rate increases. Blue increases as growth rate decreases. The scale for death rate and growth rate are relative to untreated growth rate. (**C)** FV and GR calculated for full parameter space in (B). **(D-E)** Examples of GRADE calculations (left) and inference of growth and death rates at individual doses (right). For each drug, doses not used in GRADE calculation are colored grey. White dot in each is a dose for which growth and death rates are highlighted. Growth rates and death rates are computed relative to the untreated cell proliferation rate. In these data the doubling time was 26.3868 hours (0.0379 cell divisions per hour). Data in (D-E) are mean +/- s.d. of 3 biological replicates. **(F-G)** GRADE stability of time. **(F)** FV vs. GR over time for ABT-737 (ABT). Data are mean +/- s.d. of four biological replicates. **(G)** Drug GRADE for ABT measured at time points listed. **(H-J)** FV and GR drug responses calculated for U2OS cells (WT, grey) or U2OS-*BAX*$^{-/-}$/*BAK*$^{-/-}$ (KO, red). BAX/BAK double knockout renders cells resistant to drugs that induce apoptotic death. Data are from Richards et al. (2020). Dose of drug represented by increasing shades of grey or red. Data are are the mean +/- s.d. of biological triplicate measurements **(H)** BH3 mimetic, ABT737, induces apoptotic death in U2OS cells. **(B)** BRD4/Bromodomain inhibitor, JQ1, induces non-apoptotic death in U2OS cells. **(C)** Nitrogen mustard/DNA alkylating chemotherapeutic, Chlorambucil, slows the proliferation rate but does not kill U2OS cells at any dose (non-lethal drug). For (A-C) drug GRADE for WT and KO cells calculated and shown below the respective plots.

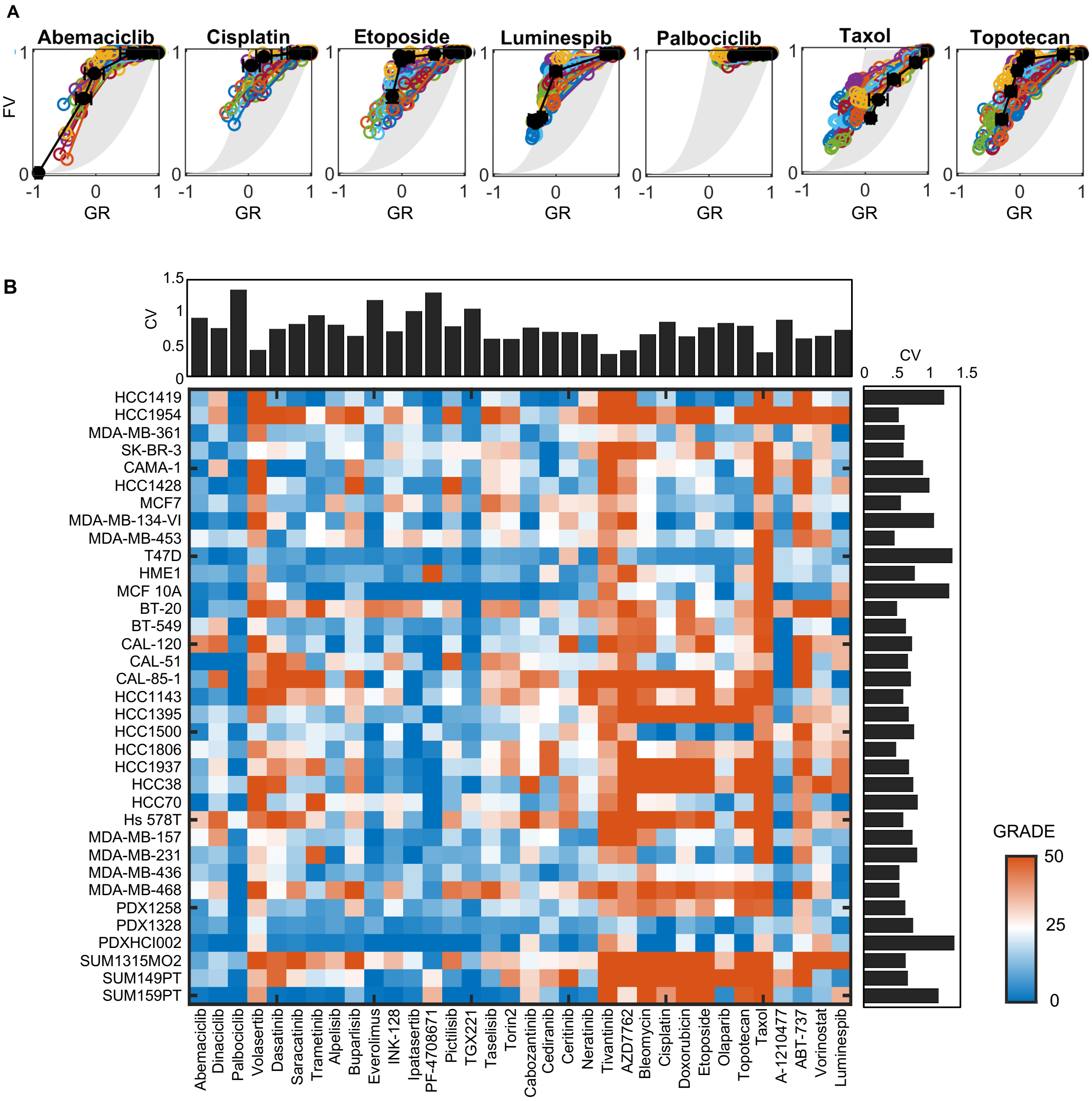

Figure S5: GRADEs for 34 drugs in 35 LINCS cell lines, Related to Figure 5. (A) GR/FV plots for 7 example drugs tested in Hafner et al. (2019), across 35 LINCS consortium cell lines. Mean GR/FV values for LINCS consortium cell lines are shown as colored dots with different doses of a drug connected by a line. U2OS is shown as a black dot and line. Error bars for U2OS are the standard deviation across 4 replicates. **(B)** Drug GRADEs for all 34 drugs tested in 35 LINCS cell lines. Drug GRADE for all drugs tested by in Hafner et al. (2019) across LINCS consortium cell lines. Heatmap colored according to GRADE. Bar plots show the coefficient of variation (CV) across all cell lines for a given drug, or across all drugs for a given cell line. The mean GRADE across all cells and drugs was 25.
